# NAD^+^ supplementation prevents STING‐induced senescence in ataxia telangiectasia by improving mitophagy

**DOI:** 10.1111/acel.13329

**Published:** 2021-03-18

**Authors:** Beimeng Yang, Xiuli Dan, Yujun Hou, Jong‐Hyuk Lee, Noah Wechter, Sudarshan Krishnamurthy, Risako Kimura, Mansi Babbar, Tyler Demarest, Ross McDevitt, Shiliang Zhang, Yongqing Zhang, Mark P. Mattson, Deborah L. Croteau, Vilhelm A. Bohr

**Affiliations:** ^1^ Laboratory of Molecular Gerontology National Institute on Aging NIH Baltimore MD USA; ^2^ Mouse Phenotyping Unit National Institute on Aging NIH Baltimore MD USA; ^3^ Electron Microscopy Core National Institute on Drug Abuse Intramural Research Program NIH Baltimore MD USA; ^4^ Laboratory of Genetics and Genomics National Institute on Aging National Institutes of Health Baltimore MD USA; ^5^ Department of Neuroscience Johns Hopkins University School of Medicine Baltimore MD USA; ^6^ Danish Center for Healthy Aging University of Copenhagen Copenhagen Denmark

**Keywords:** Ataxia Telangiectasia, mitophagy, Nicotinamide riboside, SASP, senescence

## Abstract

Senescence phenotypes and mitochondrial dysfunction are implicated in aging and in premature aging diseases, including ataxia telangiectasia (A‐T). Loss of mitochondrial function can drive age‐related decline in the brain, but little is known about whether improving mitochondrial homeostasis alleviates senescence phenotypes. We demonstrate here that mitochondrial dysfunction and cellular senescence with a senescence‐associated secretory phenotype (SASP) occur in A‐T patient fibroblasts, and in ATM‐deficient cells and mice. Senescence is mediated by stimulator of interferon genes (STING) and involves ectopic cytoplasmic DNA. We further show that boosting intracellular NAD^+^ levels with nicotinamide riboside (NR) prevents senescence and SASP by promoting mitophagy in a PINK1‐dependent manner. NR treatment also prevents neurodegeneration, suppresses senescence and neuroinflammation, and improves motor function in *Atm^−/−^* mice. Our findings suggest a central role for mitochondrial dysfunction‐induced senescence in A‐T pathogenesis, and that enhancing mitophagy as a potential therapeutic intervention.

## Graphical abstract

The underlying cause in most A‐T cases is complex, likely reflecting risks premature aging, multiple genetic factors, and non‐genetic (e.g., environmental, lifestyle/behavioral, and metabolic) factors. These factors can directly/indirectly cause mitophagy defects, leading to accumulation of damaged mitochondria, a major feature of ATM‐deficient animals and A‐T patients. Damaged mitochondria accumulate and release DNA into cytoplasm, which activates STING‐induced glial responses, senescence and SASP. Mitophagy induction by NR treatment maintains a healthy mitochondrial pool and prevents STING activation through efficient clearance of dysfunctional mitochondria and maintains a healthy brain.

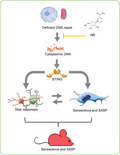

## 1 INTRODUCTION

ATM kinase, encoded by the ataxia telangiectasia‐mutated (ATM) gene, is a master regulator of the DNA double‐strand break repair responses and contributes to cellular redox balance (Guleria & Chandna, 2016; Lee & Paull, 2020). In response to DNA damage, ATM is activated by autophosphorylation and then phosphorylates several downstream proteins that enhance DNA repair (Shiloh, 2014). In humans, loss of the ATM gene results in ataxia telangiectasia (A‐T), a rare inherited autosomal‐recessive genetic disease characterized by cancer predisposition, radiosensitivity, neurodegeneration, sterility, and acquired immune deficiency. A‐T patients also suffer from a variety of inflammatory phenotypes, likely associated with a failure of both T‐ and B‐cell development (Chessa et al., 2016; Härtlova et al., 2015). While some major features of A‐T reflect inefficient DNA double‐strand break repair, the molecular basis for the cerebellar atrophy in A‐T is poorly understood.

Tissues affected in many age‐related diseases display an accumulation of senescent cells with distinct phenotypes characterized by cell cycle arrest, resistance to apoptosis, and the secretion of proinflammatory molecules (Munoz‐Espin & Serrano, 2014). It was reported that activated microglia and astrocytes accumulate in the cerebellum of *Atm^−/−^* mice (Liu et al., 2005; Song et al., 2019). Additionally, ATM‐deficient cells and animals undergo senescence and exhibit SASP. It is becoming increasingly evident that persistent DNA damage and senescence are linked. Unresolved DNA damage can impair mitochondrial function, promote disease development, and accelerate aging, as reported in A‐T (Valentin‐Vega et al., 2012). Further, ATM dysfunction causes an accumulation of cytoplasmic DNA that triggers an antiviral immune response in the brain through STING activation (Song et al., 2019). These results suggest that ATM dysfunction involves a potentially important link between STING activation and senescence phenotypes in A‐T.

Loss of mitochondrial function can promote age‐related decline in the function of many post‐mitotic cells, including muscle cells and neurons (Fang et al., 2019; Herbst et al., 2007). Multiple lines of evidence point to mitochondrial dysfunction as a component of A‐T features (Stagni et al., 2018; Valentin‐Vega et al., 2012). Dysfunctional mitochondria can induce cellular senescence in culture (Wiley et al., 2016) and in animals (Dai et al., 2010). However, little is known about the connections between senescence, neuroinflammation, and mitochondrial dysfunction. NAD^+^ which is a key cellular metabolite is critical for mitochondrial function and is deficient in ATM deficient neurons (Fang et al., 2016; Zhang et al., 2016).

In this study, we have used cross species analyses to investigate senescence phenotypes and mitochondrial dysfunction in A‐T fibroblasts, ATM‐deficient neural cells and mice. We found that loss of ATM causes an accumulation of cytoplasmic DNA, partly released from damaged mitochondria, triggering a STING‐dependent senescence phenotype in the brain and *in vitro*. Boosting NAD^+^ levels with NR removes damaged mitochondria by stimulating mitophagy and prevents senescence and SASP in A‐T models. Our findings link the neurological symptoms of A‐T directly to senescence, SASP, and impaired mitochondrial homeostasis.

## 2 RESULTS

### 2.1 Senescence phenotypes and mitochondrial dysfunction in A‐T fibroblasts

**FIGURE 1:**
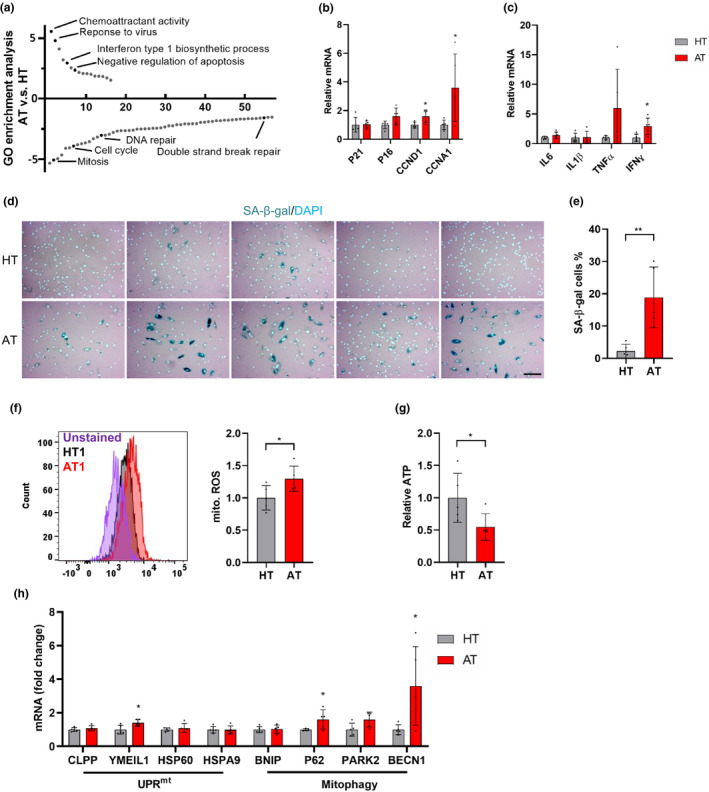
Senescence phenotypes and mitochondrial dysfunction in A‐T fibroblasts. (a) Go term analysis demonstrates up‐ and downregulated signaling pathways in A‐T fibroblasts (AT1‐4), compared with healthy controls (HT1‐4). The specific pathways related to senescence phenotypes in A‐T are highlighted in dark. X‐axis is the pathway number. (b, c) Relative genes expression for senescence (b), and SASP (c) between AT cells and HT cells. P16, *p* = 0.062. (d, e) The representative SA‐β‐gal staining images (d) and quantification of the percentage of SA‐β‐gal cells (e). Scar bar, 100 µm. (f) Mitochondrial superoxide was measured by Mitosox. The representative Mitosox staining in HT1 and AT1 (left) and quantification of the Mitosox (right) in AT compared with HT cells. (g) Cellular ATP levels in AT and HT cells. (h) Relative gene expression for UPR^mt^ and mitophagy genes in AT compared with HT cells. Data are normalized to GAPDH mRNA transcript levels. Each experiment shows the average of results obtained in triplicate for each cell line. All statistical significance was calculated by Student's t test. Data are shown as mean ± SD. *p < 0.05, **p < 0.01, ***p < 0.001

Senescent cells, characterized by sustained cell cycle arrest and production of SASP, accumulate with age and in age‐related diseases (Baker & Petersen, 2018). To verify whether senescence phenotypes occur in A‐T patient primary cells, we performed unbiased gene expression microarray analysis from A‐T patients (AT1‐4) and age‐ and sex‐matched healthy control subjects (HT1‐4). Analysis of gene ontology (GO) terms revealed upregulated immune responses, negative regulation of apoptosis, and downregulated cell cycle pathways in A‐T primary fibroblasts (Figure 1a). This result suggests that cellular senescence occurs in A‐T patient fibroblasts, which is consistent with previous findings (Herbig et al., 2004). Similarly, genes with the largest z‐ratio changes are shown in Figure S1A, and the most upregulated genes, such as IFI6, PTGER4, LINC01013, IFIT1, and IL17D, are related to SASP signaling and immunity. To confirm the microarray data, we evaluated the gene expression of senescence and SASP markers by qPCR. Compared with 5 control fibroblasts (HT cells), 5 A‐T patient fibroblasts (AT cells) displayed significantly higher expression of senescence and SASP‐associated genes (Figure 1b,c). To further investigate senescence in AT cells, we performed a senescence‐associated beta‐galactosidase (SA‐β‐gal) assay. As shown in Figure 1d,e, an increased percentage of SA‐β‐gal‐stained cells is observed in AT compared with HT cells. As neurodegeneration is one of the major problems for A‐T patients, we expanded our study by using the neuroblastoma SH‐SY5Y cell line. Knockdown of ATM in SH‐SY5Y cells resulted in increased protein levels of P21 and γH2AX, and a higher percentage of SA‐β‐gal‐stained cells compared with control (shCtrl) cells (Figure S1B,C).

Recent studies reported that cellular senescence is associated with mitochondrial defects (Wiley et al., 2016). We therefore investigated the effects of ATM dysfunction on mitochondrial function in AT cells. We found that AT cells displayed higher mitochondrial reactive oxygen species (mito. ROS) (Figure 1f) and lower ATP levels (Figure 1g) compared with HT cells, while the mitochondrial membrane potential (mito.TMRM) and mitochondrial content (mito. Green) were slightly, but not significantly increased (Figure S2A,B).

We next examined the gene expression of mitochondrial unfolded protein response (UPR^mt^) and of mitophagy pathways, which are critical for the maintenance of mitochondrial homeostasis (Pickles et al., 2018). Our results showed that gene expression patterns related with UPR^mt^ and mitophagy in AT cells are higher than in HT cells (Figure 1h). Immunoblotting of total lysates from AT cells showed increased phosphorylated AMP‐activated protein kinase (*p*‐AMPK) and decreased mitophagy‐related proteins compared with HT cells (Figure S2C,D). These findings combined with the increased mitochondrial content suggest that defective mitochondrial function and impaired mitophagy occur in AT cells. Evidence of mitochondrial dysfunction was also found in ATM knockdown SH‐SY5Y cells with higher mitoROS, mitochondrial content, and membrane potential (Figure S2E).

### 2.2 NR ameliorates STING‐mediated senescence in A‐T fibroblasts

**FIGURE 2:**
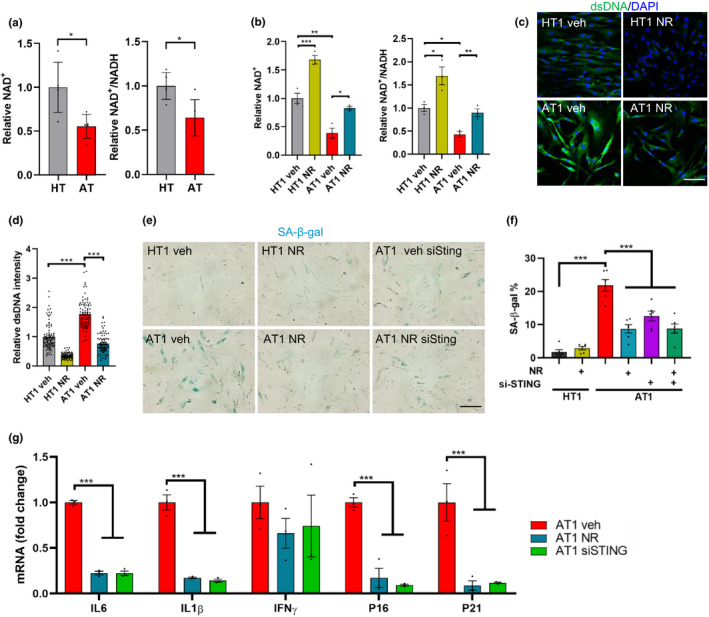
NR treatment and STING inhibition attenuates senescence in AT1 fibroblasts. (a) The relative NAD^+^ level and NAD^+^/NADH ratio in HT and AT cells. Data are shown as mean ± SD. (b) The relative NAD^+^ levels and relative NAD^+^/NADH ratio in HT1 and AT1 with or without 10 days NR treatment. (c, d) Immunofluorescence microscopic analysis of dsDNA (green) in HT1 and AT1 cells (c) and quantification of density of cytoplasmic dsDNA (d). Scar bar, 100 µm; n = 85–95 cells per group. (e, f) Representative SA‐β‐gal staining images of HT1 and AT1 cells with NR treatment for 10 days or with siSTING transfection for 48 h (e). Scar bar, 200 µm. Quantification of percentage of SA‐β‐gal‐positive cells, n = 6 cultures per group (f). (g) qPCR analysis for senescence markers in HT1 or AT1 cells treated with vehicle, NR, or siSTING, including IL6, IL1β, IFNγ, P16 and P21. n = 3 per group. Data are shown as mean ± SEM. *p < 0.05, **p < 0.01, ***p < 0.001 by two‐tailed Student's t test or one‐way ANOVA

The ATM protein kinase plays an important role in repair of DNA double‐strand breaks. DNA repair defects due to ATM deficiency are associated with persistent DNA damage and persistent activation of poly‐ADP‐ribose polymerase 1 (PARP1), which results in reduction of cellular NAD^+^ levels. As NAD^+^ plays important roles in DNA repair, mitochondrial homeostasis, senescence, and longevity in cells, worms, and mice (Fang et al., 2017; Wiley et al., 2016), we examined here whether the NAD^+^ level and NAD^+^/NADH ratio were indeed disturbed in the primary AT cells. As shown in Figure 2a, NAD^+^ levels and the NAD^+^/NADH ratio in AT cells were both lower than those in the HT cells. Because all AT cells had different growth rates and were difficult to maintain in a similar passage, we chose the sex‐ and age‐matched HT1 and AT1 as the representative cell model in subsequent experiments. These cells have been widely used in previous studies (Davis & Kipling, 2009; Hande et al., 2001). We then investigated whether boosting intracellular NAD^+^ levels with NR could prevent senescence. The result in Figure 2b shows that NR supplementation for 10 days restored NAD^+^ levels and upregulated the NAD^+^/NADH ratio in AT1 cells.

It has been previously reported that ATM‐deficient cells release damaged DNA into the cytoplasm where it can activate the cytoplasmic DNA sensing STING‐mediated pathway, which triggers the production of SASP factors, thereby promoting senescence phenotypes (Härtlova et al., 2015; Song et al., 2019). We determined whether AT1 cells have higher cytoplasmic DNA than HT1 cells and further investigated whether restoration of NAD^+^ levels could reduce cytoplasmic DNA in AT1 cells. As observed in Figure 2c,d, dsDNA in the cytoplasm was significantly higher in AT1 fibroblasts than in HT1 cells and the elevated cytoplasmic dsDNA level in AT1 cells was significantly reduced to a level close to the HT1 cells after NR treatment. Based on this result, we further asked whether NR or STING inhibition could alleviate senescence phenotypes in AT1 cells. The results in Figure 2e,f showed that NR markedly reduced SA‐β‐gal‐positive cells in AT1 cells and significantly decreased expression of senescence and SASP genes (Figure 2g). Additionally, AT1 cells treated with STING‐siRNA (siSTING) displayed reduced senescence and SASP phenotypes in AT1 cells (Figure 2e–g). This suggests that activated STING by cytoplasmic dsDNA plays a key role in senescence phenotypes and that NR may alleviate senescence via inhibition of the STING pathway.

### 2.3 NR prevents senescence by enhancing mitophagy

**FIGURE 3:**
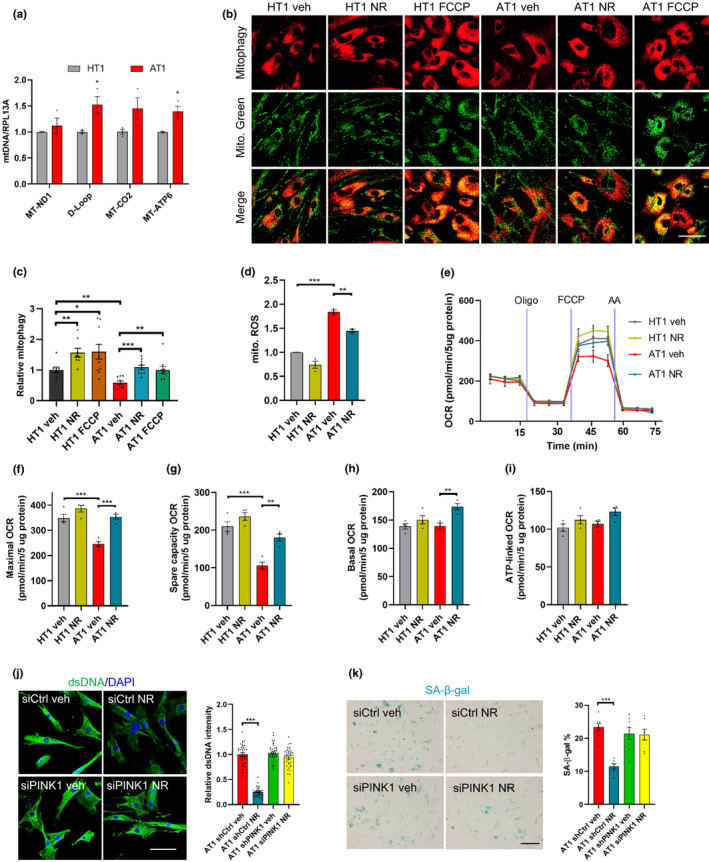
NR attenuates senescence via enhancing mitophagy. (a) DNA was harvested from cytoplasmic and nuclear fractions of HT1 and AT1, and mtDNA/RPL13A was analyzed by qPCR. n = 3 cultures per group. (b, c) Detection of mitophagy using an mitophagy dye (Dojindo Molecular Technologies) in AT1 and HT1 with or without NR treatment (1 mM, 24 h) and FCCP (30 µM, 24 h). Mitophagy was calculated by normalizing the mitophagy pixels to the mito. Green pixels. Confocal microscopy images in (b) and quantification of mitophagy in (c). Scar bar, 100 µm; n = 8–10 cultures per group. (d) Mitochondrial ROS was measured by flow cytometry in HT1 and AT1 cells. n = 3 per group. (e–i) OCR in HT1 and AT1 cells with or without NR treatment (e). Quantification of maximal (f), spare respiratory capacity (g), basal (h), and ATP‐linked OCR (i). Data are normalized to protein levels. N = 4 per group. (j) Immunofluorescence microscopic analysis of dsDNA (green) in HT1 and AT1 cells (left) and quantification of density of cytoplasmic dsDNA (right). Scar bar, 100 µm; n = 35–40 cells per group. (k) Representative images of SA‐β‐gal staining in HT1 and AT1 cells (left) and quantification of percentage of SA‐β‐gal staining (right). Scar bar, 200 µm. n = 3 cultures per group. Data are shown as mean ± SEM. *p < 0.05; **p < 0.01; ***p < 0.001 by two‐tailed Student's t test or one‐way ANOVA

As mitochondrial dysfunction was evident in A‐T cells, we further explored the possibility that loss of ATM induces mtDNA release into the cytoplasm since liberated mtDNA also induces STING‐dependent innate immune activation (Sun et al., 2017). AT1 and HT1 cells were fractionated into cytoplasmic and nuclear fractions to quantify the level of DNA containing specific mitochondrial (MT‐ND1, D‐loop, MT‐CO2, and MT‐ATP6) and nuclear (RPL13A) genes by qPCR (Aarreberg et al., 2019). Results in Figure 3a indicated that AT1 cells have a significant enrichment of mtDNA in the cytoplasmic fraction when compared with HT1 by 1.2‐~1.5‐fold depending on the genes. This indicates that the release of mtDNA from damaged mitochondria into cytoplasm may serve as a possible trigger for inflammation and senescence in AT1 cells.

The increase of cytoplasmic mtDNA in AT1 cells indicated a possible deficiency in removing damaged mitochondria, which in part is regulated by mitophagy, the selective degradation of damaged or dysfunctional mitochondria by autophagy (Pickles et al., 2018). By employing a mitophagy Dojindo dye, which allows the detection mitochondria in an acidic environment, we found that the mitophagy level in AT1 cells was indeed lower than in the HT1 cells under basal conditions (Figure 3b,c). NR and FCCP treatment significantly increased mitophagy levels in both HT1 and AT1 cells (Figure 3b,c).

Consistently, mitochondrial functions were restored in AT1 cells after NR treatment, as manifested by lower levels of mitochondrial ROS (Figure 3d) and increased oxygen consumption rates (OCR) (Figure 3e–i). Notably, maximal (Figure 3f) and spare capacity OCR (Figure 3g) were significantly impaired in AT1 compared with HT1, and these indexes were significantly improved after NR treatment. There were no significantly differences between AT1 cells and HT1 cells in basal respiration and ATP‐linked OCR (Figure 3h,i).

We further investigated whether the effect of NR on senescence was regulated through mitophagy by knocking down the PTEN‐induced kinase1 (PINK1) gene, which is important in mitophagy regulation in ATM models (Fang et al., 2016). The results showed that NR failed to reduce the dsDNA intensity (Figure 3j), SA‐β‐gal‐positive cells (Figure 3k), and gene expression of senescence and SASP markers (Figure S3) in AT1 siPINK1 cells, indicating that mitophagy is required for the effects of NR in reducing senescence.

### 2.4 NR improves mitochondrial functions and reduces cytoplasmic dsDNA in neural cells

**FIGURE 4:**
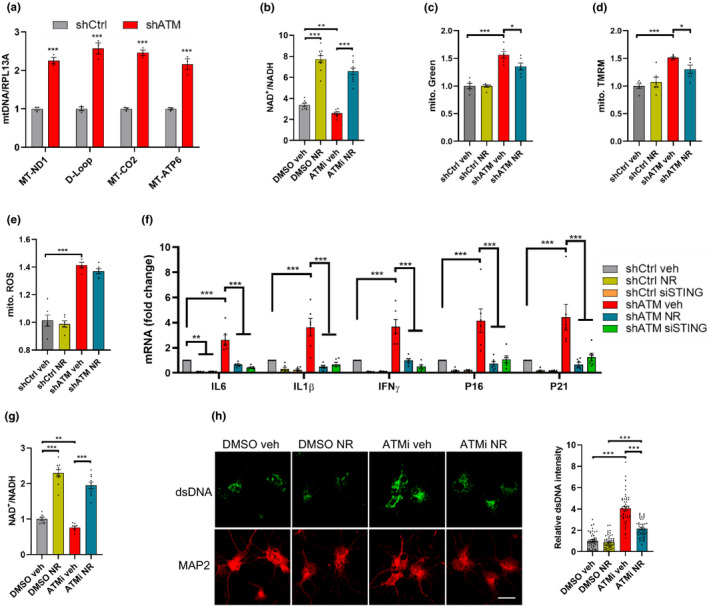
NR ameliorates mitochondrial dysfunction and cytoplasmic dsDNA in neural cells. (a) DNA was harvested from cytoplasmic and nuclear fractions of ATM knockdown or control SH‐SY5Y cells and mtDNA/RPL13A was analyzed by qPCR. n = 3 cultures each group. (b) The ratio of NAD^+^/NADH in shCtrl and shATM SH‐SY5Y cells. (c–e) Mitochondrial content (c), mitochondrial membrane potential (d), and mitochondrial ROS (e) were measured by flow cytometry in SH‐SY5Y cells. n = 6 cultures per group. (f) qPCR analysis for senescence markers in shCtrl‐ or shATM SH‐SY5Y cells treated with vehicle, NR, or siSTING, including IL6, IL1β, IFNγ, P16, and P21. n = 8 cultures per group. (g) The ratio of NAD^+^/NADH were analyzed in DMSO‐ or ATMi‐neurons with or without NR treatment. n = 8–10 cultures per group. (h) Immunofluorescence microscopic analysis of dsDNA (green) and neuronal maker MAP2 (red) (left) and quantification of density of dsDNA (right). Scar bar, 10 µm; 47–54 neurons were analyzed for each group. Data are shown as mean ± SEM. *p < 0.05; **p < 0.01; ***p < 0.001 by two‐tailed Student's t test or one‐way ANOVA

Because neurons are the cell type that degenerate in A‐T, we next investigated the effects of NR in ATM‐deficient SH‐SY5Y neuronal cells. Consistent with the results observed in AT1 cells, there was a significant enrichment of mtDNA in the cytoplasmic fraction from shATM SH‐SY5Y compared with control cells, by ~2.5‐fold (Figure 4a). The NAD^+^/NADH ratio was also decreased in shATM SH‐SY5Y cells and increased after NR treatment (Figure 4b). Further, NR decreased the mitochondrial content (Figure 4c) and mitochondrial membrane potential (Figure 4d) in ATM knockdown SH‐SY5Y cells while no significant change in mitochondrial ROS (Figure 4e, *p* = 0.122) was observed after NR treatment. Furthermore, both NR treatment and STING knockdown normalized expression of senescence genes (Figure 4f), which is consistent with the results in AT1 cells.

We also employed primary neurons from embryonic rats and exposed them to ATM inhibitor (ATMi, KU‐60019, 1 µm, Figure S4A), as described previously (Song et al., 2019). We observed that ATMi‐treated cells exhibited a lower NAD^+^/NADH ratio compared with DMSO‐treated cells. NR supplementation upregulated the NAD^+^/NADH ratio and NAD^+^ levels (Figure 4g). Additionally, analysis of mitochondrial respiration revealed significantly reduced basal respiration, and a trend toward decreased maximal respiration in ATMi neurons compared with DMSO‐treated neurons, while NR markedly increased basal, maximal and ATP‐linked OCR in ATMi‐neurons (Figure S4B,C). Furthermore, cytoplasmic dsDNA was significantly higher in ATMi neurons than DMSO neurons and was reduced after NR treatment (Figure 4h). ATMi‐treated neurons had significantly increased DNA damage marker, 53BP1, compared with DMSO neurons (Figure S4D), consistent with the defective DNA damage response found in the A‐T brain (Biton et al., 2006) and in *Atm^−/−^* mice (Li et al., 2013). Notably, NR substantially diminished the intensity of 53BP1 in ATMi‐treated neurons (Figure S4D), which is consistent with previous reports that DNA repair is enhanced after NAD^+^ supplementation through NR (Fang et al., 2016). Together, these findings suggest that NR treatment can restore mitochondrial dysfunction and impaired DNA damage repair induced by loss of ATM.

### 2.5 NR prevents neurodegeneration and senescence in *Atm^−/−^* mice

To further understand the underlying features of A‐T phenotypes in *Atm^−/−^* mice, we extracted mRNA from the most affected region in the brain, the cerebellum, and performed microarray analysis which provides an unbiased means to identify altered genes and signaling pathways. Young adult *Atm^–/–^* mice and their age‐ and sex‐matched wild‐type (WT) littermates were treated with 12 mM NR in their drinking water, starting from 1.5‐month‐old for 2 months. Heat map clustering analysis of differentially expressed genes (Figure S5A) and principal component analysis (PCA, Figure S5B) revealed a separation between WT veh and *Atm^−/−^* veh, and NR treatment led to a shift of the *Atm^−/−^* transcriptomic profile toward the WT veh profile. Significantly changed GO terms were defined as those displaying an absolute *z*‐score of at least 1.5, a *p*‐value <0.05, and a false‐discovery rate (FDR) ≤0.3 and having at least three changed genes in each term in at least one comparison. Among the genes with the largest enrichment score and significant changes in the comparison between WT veh and *Atm^−/−^* veh were *Lrrc49*, *Hmmr*, *Pign*, *Cyp2t4*, *Dzip1*, *SpacA6*, *ST14*, and *Cd209d* genes, which are related to inflammatory pathways (Table S1). Comparing with WT veh, the WT NR group had increased immune‐related genes, Pstpip2, Atp6v1g3, and Adgrg3 (Table S1).

Individuals with A‐T have been shown to develop immunodeficiency, sensitivity to virus infection, and systemic inflammation (McKinnon, 2004). Consistent with our genes expression profiles, enrichment scores of *Atm^−/−^* versus WT revealed upregulation of the humoral immune response and response to virus (Figure S5C) and NR treatment alleviated these alterations (Figure S5D). In addition, we found that the terms of neuronal and synaptic functions were downregulated in *Atm^−/−^* mice compared with the WT mice, and that NR treatment restored expression of genes in neuronal function in *Atm^−/−^* mice (Figure S5E). These findings suggest that NAD^+^ supplementation profoundly impacts A‐T pathology via multiple mechanisms.

**FIGURE 5:**
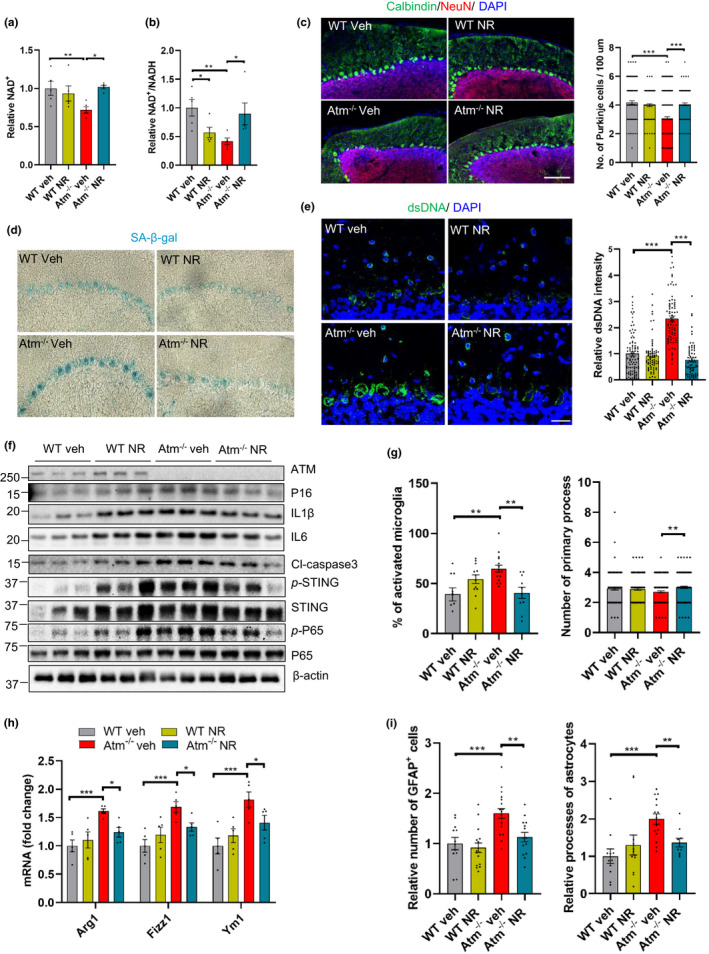
NR inhibits neurodegeneration and senescence phenotypes in *Atm^−/−^* mice. (a, b) Cerebellar tissue of WT and *Atm^−/−^* mice treated with vehicle or NR were measured for relative NAD^+^ (a) and ratio of NAD^+^/NADH (b). n = 5 mice per group. (c) Representative images of NeuN‐ and Calbindin‐stained Purkinje cells show the effects of NR (left) and quantification of the number of Purkinje cells per 100 µm (right). Scale bar, 100 μm. n = 4 mice for each group. (d) Representative images of SA‐β‐gal staining in cerebellum of WT and *Atm^−/−^* mice with vehicle‐ or NR treatment for 2 months. (e) Immunofluorescence microscopic analysis of dsDNA (green) in cerebellum (left) and quantification of density of cytoplasmic dsDNA (right). Scar bar, 50 µm; n = 4 mice per group. 70–90 cells were analyzed in each group. (f) Senescence, SASP, and cell death markers were analyzed by immunoblotting in cerebellar tissue, including ATM, P16, IL1β, IL6, cleaved caspase‐3 (Cl‐caspase3), *p*‐STING, STING, *p*‐P65, and P65 protein levels. Quantification of protein levels are shown in Figure S6A. n = 3 mice per group. (g) Quantification of percentage (left) and number of primary process (right) of activated microglia in vehicle‐ or NR‐treated WT and *Atm^−/−^* mice brain immunostained with microglia marker anti‐Iba1. Representative images in Figure S6D. n = 4 mice per group. (h) Relative genes expression of M2 microglia markers Arg1, Fizz1, and YM1 in cerebellar tissue. n = 5 mice per group. (i) Quantification of relative number of astrocytes near Purkinje cell layer in vehicle‐ or NR‐treated mice (left). Relative astrocytes processes at the molecular layer of cerebellum (right). n = 4 mice per group. Representative images in Figure S6D. Data are shown as mean ± SEM. *p < 0.05; **p < 0.01; ***p < 0.001 by two‐way ANOVA

As cerebellar Purkinje cells are the most affected neurons in ATM‐deficient animals and in A‐T patients (Crawford et al., 2006; Genik et al., 2014), we examined the number of Purkinje cells and the potential benefits of NAD^+^ supplementation in *Atm^–/–^* mice. Consistent with the findings in the A‐T cells, *Atm^−/−^* mice showed a significantly reduced NAD^+^ level (Figure 5a) and NAD^+^/NADH ratio compared with WT mice (Figure 5b) and NR treatment reversed these alterations in *Atm^−/−^* mice (Figure 5a,b). Consistent with a previous study (Li et al., 2013), *Atm^−/−^* mice exhibited a significant reduction of Purkinje cell counts compared with WT mice, and NR attenuated the loss of Purkinje cells in the *Atm^−/−^* mice (Figure 5c).

Senescent cells accumulate in the nervous system with aging and in neurodegenerative diseases and may predate the appearance of neurodegeneration or aggravate its course (Kritsilis et al., 2018). To measure senescence *in vivo*, we stained free‐floating brain sections from 3.5‐month‐old *Atm^−/−^* mice with or without NR treatment for SA‐β‐gal activity. Based upon the size and morphological features the SA‐β‐gal‐positive cells, we identified that SA‐β‐gal‐positive cells are located in the Purkinje cell layer and that they were much more prominent in *Atm^−/−^* mice compared with WT mice. The SA‐β‐gal staining intensity was reduced in NR‐treated *Atm^−/−^* mice (Figure 5d).

We also observed that the dsDNA level in cerebellar Purkinje cell layer from *Atm^−/−^* mice was significantly higher than in WT mice (Figure 5e), which is consistent with a previous study (Song et al., 2019). Importantly, NR treatment dramatically decreased dsDNA in the cerebellar tissue of *Atm^−/−^* mice (Figure 5e).

We next sought to investigate whether STING was activated due to the accumulation of cytoplasmic DNA in *Atm^−/−^* mice. Remarkably, in the cerebellar lysates of *Atm^−/−^* mice, the levels of phosphorylated‐STING (*p*‐STING) were significantly higher than in WT mice (Figures 5f and S6A), as previously reported (Härtlova et al., 2015). Additionally, P65 phosphorylation (*p*‐P65) was increased in *Atm^−/−^* mice, suggesting activation of NF‐κB pathway (Figures 5f and S6A). Both *p*‐STING and *p*‐P65 protein levels were significantly reduced in *Atm^−/−^* mice after NR treatment (Figures 5f and S6A). Besides P65, STING was reported to associate with the NLRP3 inflammasome and with IRF3 activation during the inflammatory response (Li et al., 2019). Here, we showed that the NLRP3 level in *Atm^−/−^* NR mice was significantly lower than that in WT mice, but there was no difference between *Atm^−/−^* veh mice and *Atm^−/−^* NR mice (Figure S6B,C). Further, the levels of Pro‐IL18, IL18, and IFNγ showed no significant difference between WT mice and *Atm^−/−^* mice (Figure S6B,C). The *p*‐IRF3/IRF3 level in WT veh mice was higher than in WT NR, *Atm^−/−^* veh and *Atm^−/−^* NR mice, and NR had no effect on the *p*‐IRF3/IRF3 level in *Atm^−/^* ^−^ mice (Figure S6B,C). This indicates that neither NLRP3 nor IRF3 are activated in senescence phenotypes, but that the NF‐κB pathway is activated.

Importantly, the senescence marker P16 and SASP markers IL1β and IL6 found to be enriched in the cerebellar tissue of *Atm^−/−^* mice were reduced after NR treatment (Figures 5f and S6A). It is interesting to note that NR treatment increased IL1β and IL6 in WT young mice (Figures 5f and S6A), indicating that super supplementation of NAD^+^ in young healthy mice, which are in good hemostasis of NAD^+^ may have unintended consequences on inflammatory pathways. In addition, consistent with our previous study (Fang et al., 2016), the increased level of the apoptotic marker, cleaved caspase‐3 in *Atm^−/−^* cerebellar tissue was also decreased after NR treatment (Figures 5f and S6A). These findings indicate that cerebellar neurons that degenerate exhibit features of senescence prior to their death in *Atm^−/−^* mice. Both cellular senescence and apoptosis are extreme responses to cellular stress and play major roles in neurodegeneration and organismal aging (Childs et al., 2014). However, the cross‐regulation between apoptosis and senescence needs further clarification.

Accumulating evidence suggests that microglia and astrocytes play roles in ATM pathology (Liu et al., 2005; Song et al., 2019). In normal brain, the “resting” state microglia possess many fine processes, which allows the cells to extensively explore their environment. Upon stimulation, however, activated microglia adopt a different morphology with a larger spherical shape, and with fewer but thicker processes (Boche et al., 2013). The links between neurodegeneration in A‐T neurons and immune responses are poorly understood. By immunostaining with an antibody against the microglial marker Iba1, we observed ~30% more activated microglia in *Atm^−/−^* mice than WT mice, and they were reduced after NR treatment (Figure 5g and S6D). Additionally, reduced microglia primary processes in the *Atm^−/−^* mice (*p* = 0.059) were significantly increased after NR treatment (Figure 5g and S6D). We then monitored the activation state of the microglia. The classical activation is known as M1 and these cells are mediators of the proinflammatory responses. The alternative activation, known as M2, is responsible for resolution and repair (Zheng & Wong, 2019). M1 activation markers (IL6, IL1β, Figure 5f and S6A) were increased in *Atm^−/−^* mice and so were M2 markers (Arg1, Fizz1 and YM1) and NR significantly reduced the M2 markers (Figure 5h).

Astrocytes are another primary glial cell critical for many important biological processes including the inflammatory response in brain (Colombo & Farina, 2016). We next determined that *Atm^−/−^* mice have significantly more reactive‐like astrocytes, as judged by positive immunostaining for glial fibrillary acidic protein (GFAP) than WT controls (Figures 5i and S6E). NR treatment reduced astrocytes in the cerebellum, as compared to non‐NR‐treated *Atm^−/−^* mice (Figures 5i and S6E). We also saw increased GFAP‐stained processes in the *Atm^−/−^* cerebellum compared with WT mice, and this was normalized by NR treatment (Figures 5i and S6E). Together, these results suggest that NR may reduce neuroinflammation by downregulating microglial and astrocyte responses.

### 2.6 NR improves mitochondrial homeostasis and motor behaviors in *Atm^−/−^* mice

**FIGURE 6:**
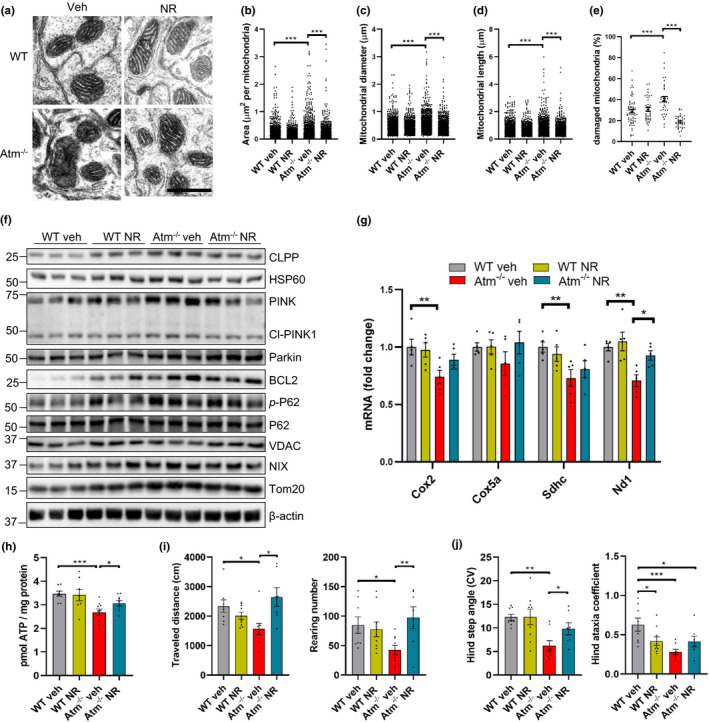
NR improves mitochondrial homeostasis and motor behaviors in WT and *Atm^−/−^* mice. (a–e) Representative electron microscopy images of cerebellum from WT and *Atm^−/−^* mice treated with NR or vehicle (a). Quantification of mitochondria area (b), diameter (c), length (d), and percentage of damaged mitochondria (e). Scar bar, 1 µm; n = 1500–1600 mitochondria from n = 4 mice per group. (f) Immunoblot analysis of cerebellum tissue from WT and *Atm^−/−^* mice after 2 months of NR treatment. Quantification of data is in Figure S7A. n = 3 per group. (g) Relative levels of mRNA implicated in the OXPHOS in cerebellar tissue. n = 5 mice for each group. (h) ATP levels in mice cerebellar tissue. n = 7–9 mice per group. (i) The total traveled distance (left) and rearing number (right) of vehicle‐ and NR‐treated mice in the open field test. (j) Hind step angle (CV) (left) and Hind ataxia coefficient (right) in DigiGait analysis test. WT veh, n = 9; WT NR, n = 10; *Atm^−/−^* veh, n = 6; *Atm^−/−^* NR, n = 7. Data are shown as mean ± SEM. *p < 0.05; **p < 0.01; ***p < 0.001 by two‐way ANOVA

We previously reported on mitochondrial dysfunction in ATM‐deficient worms and mice (Fang et al., 2016). We reasoned that one possible signal connecting NR treatment to reduced senescence phenotypes could be the improvement of mitochondrial homeostasis. To uncover the cellular and molecular causes of mitochondrial dysfunction and the accumulation of damaged mitochondria in *Atm^−/−^* cerebellum, we examined mitochondrial morphology in Purkinje cells from WT and *Atm^−/−^* mice. Purkinje cells in *Atm^−/−^* mice displayed altered mitochondrial morphology characterized by increase of disorganized and larger mitochondria compared with WT mice (Figure 6a–e). Notably, damaged mitochondria were reduced in Purkinje cells of *Atm^−/−^* mice treated with NR (Figure 6a–e).

As damaged mitochondria are characterized by impaired mitochondrial biogenesis, metabolism, and dynamics, we measured mitochondrial functions by assaying oxidative phosphorylation (OXPHOS) components by qPCR and mitophagy‐related proteins by immunoblot in cerebellar tissues. Compared with WT mice, several UPR^mt^ and mitophagy‐related proteins were upregulated in *Atm^−/−^* mice (Figures 6f and S7A), whereas OXPHOS genes were downregulated (Figure 6g). NR treatment normalized the levels of these parameters in *Atm^−/−^* mice (Figure 6f,g and S7A). To determine whether NR impacts mitochondrial bioenergetics in *Atm^−/−^* cells, we assessed the OCR in *Atm^−/−^* mouse embryo fibroblasts (MEFs). Notably, a significantly impaired maximal and spare capacity OCR were observed in *Atm^−/−^* MEFs, whereas NR treatment markedly increased the basal, maximal respiration, and spare capacity OCR (Figure S7B,C). This suggests that there is increased mitochondrial stress in *Atm^−/−^* mice and that compromised mitophagy fails to remove damaged mitochondria during A‐T disease progression while NR could normalize mitochondrial defects in *Atm^−/−^* mice. Consistently, the ATP levels were lower in cerebellum of *Atm^−/−^* mice than WT mice and increased after NR treatment (Figure 6h). In summary, our results demonstrate that NR treatment normalizes mitochondrial function in *Atm^−/−^* models.

Although ATM‐deficient mice do not recapitulate the profound ataxia in A‐T, researchers have shown reduced motor coordination and abnormal walking patterns compared with wild‐type mice (Li et al., 2013). Therefore, to test the impact of NR on motor functions, we conducted behavioral analyses in the *Atm^−/−^* mice with NR treatment for 2 months. In the open field test, the traveled distance, ambulatory time, rearing, and jumping parameters were significantly decreased in *Atm^−/−^* mice compared with WT. NR treatment reversed these deficient behaviors in *Atm^−/−^* mice (Figures 6i and S8A,B). Furthermore, DigiGait analysis, which generates numerous indices of gait dynamics and posture, showed that *Atm^−/−^* mice had significantly decreased hind step angel (CV) compared with the WT mice, which could be normalized by NR (Figure 6j). Further, the hind ataxia coefficient, an index of step‐to‐step variability, was significantly lower in *Atm^−/−^* mice than WT and NR treatment had no significant effect in *Atm^−/−^* mice (Figure 6j). The fore ataxia coefficient and step angle also did not show a difference between WT and *Atm^−/−^* mice (Figure S8C,D). These results indicate that hind legs are implicated in the impaired motor behavior in *Atm^−/−^* mice and that it could be partially improved by NR treatment.

## 3 DISCUSSION

In this study, we demonstrate that disturbed mitochondrial homeostasis plays an important role in neurodegeneration, senescence, and brain dysfunction in ATM‐deficient mice. Notably, we show that STING is partly activated by cytoplasmic mtDNA, released from damaged mitochondrial, and this may exacerbate senescence and SASP. Notably, boosting NAD^+^ improves motor functions and prevents neuroinflammation and senescence through enhancing mitochondrial function, reducing cytoplasmic DNA and preventing activation of STING in ATM models.

Senescence phenotypes are associated with mitochondrial dysfunction, and compromised mitophagy has been considered as one of the major hallmarks of neurodegenerative diseases (Baker & Petersen, 2018; Cirotti et al., 2020, 2020). Inflammatory cytokines play an essential role in the initiation and maintenance of cellular senescence and are responsible for triggering an innate immune response that clears the senescent cells. A‐T patients manifest a variety of inflammatory and autoimmune syndromes (McKinnon, 2004). Notably, the major cause of mortality and morbidity in A‐T patients is respiratory infections, at least in part, due to T‐ and B‐cell deficiencies (Song et al., 2019). Clinical studies have demonstrated that A‐T patients have systemic inflammation and anti‐inflammatory betamethasone treatments can suppress the severity of the neurological symptoms in A‐T (Giardino et al., 2013; Menotta et al., 2012). Similarly, inhibition of ATM was recently shown to result in increased inflammatory responses in glial cells, which in turn drives neurodegeneration in Drosophila (Petersen et al., 2012) and mice (Song et al., 2019). Together, these results demonstrate a hitherto underappreciated role of inflammatory responses in the clinical manifestations of A‐T and this is likely connected to the upregulation of the STING pathway. Here, we observed accumulation of cytoplasmic dsDNA *in vivo* and *in vitro* (Figures 2c, 4h and 5e), which stimulates the STING pathway. Activation of STING subsequently initiates a robust proinflammatory response and senescence, associated with the deficient health span in ATM‐deficient mice (Figures 5, 6). Senescence phenotypes are rescued by concurrent loss of STING, suggesting that STING is a central regulator of this phenotype (Figures 2e‐g, 4f,5f and S6A).

The reduction in cellular and/or tissue NAD^+^ pool accelerates multiple aging features (Fang et al., 2017), ultimately leading to a loss of mitochondrial homeostasis and senescence phenotypes in A‐T. It is known that cytoplasmic mtDNA from damaged mitochondria can also induce STING‐dependent inflammatory mediator activation (Aarreberg et al., 2019; Sun et al., 2017), which in turn contributes to senescence phenotypes. It is thus important to explore the possibility of improving mitochondrial quality to inhibit senescence, SASP, and neurodegeneration in A‐T. Recent studies show that mitochondrial stress can elevate levels of numerous proinflammatory cytokines and of senescence (West et al., 2015; Wiley et al., 2016). This suggests that mitochondrial dysfunction is implicated in senescence. Here, we demonstrate that loss of ATM induces impaired mitophagy (Figure 3b,c and S2C) and consequently promotes release of mtDNA into the cytoplasm (Figures 3a and 4a). Accumulation of cytoplasmic mtDNA activates the STING‐induced senescence phenotypes. By boosting cellular concentration of NAD^+^, the accumulation of cytoplasmic dsDNA is suppressed due in part to the activation of mitophagy which stimulates the clearance of damaged mitochondria. We further demonstrated that NR failed to prevent senescence following inhibition of the mitophagy protein, PINK1 (Figures 3j,k and S3A). These findings suggest that NR prevents senescence via enhancing mitophagy in A‐T models.

However, it is noteworthy that in addition to the regulation of senescence by mitophagy, NR may also bring health benefits to the *Atm^−/−^* mice through multiple other mechanisms. Some studies have reported abnormal phenotypes of astrocytes and microglia in *Atm^−/−^* mice (Liu et al., 2005; Song et al., 2019), which release inflammatory cytokines and promote neurodegeneration. Here, we found that NR treatment down regulates the activation of astrocytes and microglia in the *Atm^−/−^* mouse brains, thus reducing neuroinflammation (Figures 5g‐i and S6D,E). Further, we had previously reported that NR can improve non‐homologous end joining (NHEJ)‐mediated double‐strand break (DSB) repair by increasing NAD^+^/SIRT1 signaling (Fang et al., 2016). NAD^+^ dependent SIRT1 is involved in DNA repair via activation of ATM and Ku70 (Chalkiadaki & Guarente, 2015). NAD^+^ can increase repair of DSBs through activation of the Ku70 and DNA‐PKcs when ATM is defective. Other NAD^+^‐dependent sirtuin members, including SIRT6 and SIRT3, also contribute to the DNA repair improvements by NAD^+^ supplementation. Consistently, we showed that the increased DNA damage in primary neurons is reduced after NR treatment (Figure S4D).

Our data suggest that NR may have different effects on WT and *Atm^−/−^* mice. We found that NR treatment reduced the levels of P16, IL1β, IL6, and *p*‐STING proteins in *Atm^−/−^* mice while it increased those proteins levels in WT mice (Figures 5f and S6A). This difference may be due to a difference in basal NAD^+^ levels in the WT mice and *Atm^−/−^* mice. Additionally, there may be age or localized drug effects on specific tissue. It has been well established that NAD^+^ level decline with age (Camacho‐Pereira et al., 2016). In our experiment, we used 1.5‐month‐old mice and treated them with NR for two months. As they are young and healthy, WT mice most likely do not have NAD^+^ decline. Further supplementation of NAD^+^ may disturb the NAD^+^ metabolome balance and have some unexpected effects. Besides, in our experiment, we only checked the affected brain region, the cerebellum; thus, we are not sure whether NR supplementation would induce similar effects in other tissues of the WT young mice and whether modulation of NR dosage locally can alleviate these responses in the cerebellum. For the *Atm^−/−^* mice, NAD^+^ was shown in a previous report (Fang et al., 2016) and in our current data (Figure 5a) to be significantly decreased when compared to WT mice. As NAD^+^ plays important roles in multiple molecular processes, including DNA repair, mitochondrial functions, and cellular senescence, supplementation of NAD^+^ is critical and beneficial in settings such as A‐T or normal aging where NAD^+^ levels are low. Whether NAD^+^ supplementation is advisable for healthy young humans remains to be determined.

In a short conclusion, our results demonstrate that neuroinflammatory and senescence phenotypes are associated with the STING pathway. Activation of STING is partly induced by cytoplasmic dsDNA released from damaged mitochondria in A‐T cells. NAD^+^ supplementation promotes removal of damaged mitochondria by mitophagy and reduces glia responses, leading to inhibition of inflammation and senescence in A‐T models. Thus, our data support the concept that targeting the maintenance of mitochondrial quality may have potential roles in the prevention of senescence and neuroinflammation in neurodegenerative diseases.

## 4 MATERIALS AND METHODS

All detailed description of methods and materials can be found online in the Supplementary files.

## CONFLICT OF INTEREST

V.A.B. has CRADA arrangements with ChromaDex but receives no personal benefit. All others declare no competing interests.

## AUTHOR CONTRIBUTIONS

B.Y., X.D., D.L.C., and V.A.B. designed experiments. B.Y. and R.M. performed animal treatment and behavior test. B.Y., Y.H, X.D., and N.W. collected the tissues from animals. B.Y., X.D., N.W., S.K., J.L., M.B., T.D., and R.K. performed Western blot, histology, IHC, qPCR, mitochondrial analysis, and cellular experiments. S.Z. performed tissue preparation and imaging for EM data. Y.Z. and D.L.C. performed microarray analysis. B.Y. and X.D. wrote and B.Y., X.D., D.L.C, M.P.M., and V.A.B. revised the manuscript. All authors contributed to writing the final manuscript.

## Supporting information

Supplementary Material

## Data Availability

The data that supports the findings of this study are available in the supplementary material.
