# Supplementary material for: NAD+ supplementation prevents STING‐induced senescence in ataxia telangiectasia by improving mitophagy
Source: Aging Cell. 2021 Mar 18;20(4):e13329. doi: 10.1111/acel.13329 (PMC8045911; doi:10.1111/acel.13329)
Supplement: Supplementary file 1 — Supplementary Material [file ACEL-20-e13329-s001.pdf]

SUPPORTING INFORMATION

Supplemental Experimental Procedures

Cell culture and treatment

Human fibroblasts were obtained from Coriell Institute (Camden, NJ). Healthy controls (HT1-5): GM07532, AG01522, GM05757, GM00316, GM08398. A-T cells (AT1-5): AG03058, GM07481, GM03487, GM03395, AG4405. Cells were cultured in DMEM supplemented with 10% FBS and 1% penicillin-streptomycin (10,000 U/ml; Thermo Fisher Scientific) at 37°C in a humidified incubator with 5% CO2/95% air. All cells at passages 20-23 were used in different assays.

Human neuroblastoma cell line SH-SY5Y (ATCC) were cultured in DMEM/F12 supplemented with 10% FBS and 1% penicillin–streptomycin (10,000 U/ml; Thermo Fisher Scientific) at 37°C in a humidified incubator with 5% CO2/95% air. Cells were plated in the density of 300,000 cells per well into a 6-well plate. After 24h seeding, cells transfected with validated lenti-shATM virus (Sigma, TRCN0000194861) or lenti-shCtrl virus (Sigma, SHC001) were selected by 1.5 ug/ml puromycin.

siSTING and SiPINK1 were purchased from Dharmacon. Knockdown experiments were performed according to manufacturer's procedures. Nicotinamide riboside/NR, 1mM were added to the cells.

Mice

The Atm heterozygous strain (B6;129S4-Atm^{tm1Bal}/J) was purchased from The Jacksons Laboratory. Atm homozygous (*Atm*$^{-/-}$) mice and their wild type (WT) littermates were used for experiments. Mice were maintained in the in a constant-temperature facility with a 12 h light/12 h dark cycle and given food and water ad libitum. Animal phenotyping experiments were carried out according to standard operational procedures (SOPs) established and validated by Transnetyx. For genotyping, primers for WT included forward primer (F): TCAAGGCATGCCATTATCTGTCTT, reverse primer (R): CTGACTGGAAACTGATCGCTACTTT; primers for mutant allele were F:

GGGCGCCCGGTTCTT, and R: CCTCGTCCTGCAGTTCATTCA. The *Atm*$^{-/-}$ mice and their age- and sex-matched littermates were used for behavioral studies. All animal experiments were performed and approved by NIA Animal Care and Use Committee.

Behaviors

For behavioral tests, 1.5-month-old mice were given NR at 3.5 mg/ml (12 mM) in their drinking water while the control groups were received only drinking water for 2 months. In the open field test, each individual mouse was placed in the center of open field box (40 cm×40 cm×40 cm) (Med Associates, Georgia, VT, USA) and recorded for 20 minutes. The tracking software (Activity monitor version 4, Med-Associates) recorded the exploratory behavior. The apparatus was cleaned with 70% ethanol before testing the next mouse. Travel distance, number and duration of jump and rearing parameters were recorded and analyzed. In Digi-gait test, mice were habituated for 5 minutes. Animals were recorded by collecting digital images at 80 frames per second with a high-speed video camera located beneath a transparent treadmill moving at a speed of 15 cm/s (Digi-Gait Imaging Systems, Boston, MA). A variety of gait parameters were analyzed R software.

NAD^+ detection

NAD^+ and NADH were measured with a commercially available NAD^+/NADH assay kit (Abcam, #ab65348) according to the manufacturer's protocol. For cells, 4 X 10^5 cells homogenized in 400 µl lysis buffer. For mice tissue, 20 mg were homogenized in 500 µl lysis buffer and clarified at 17,000 g for 5 min at 4 °C. Supernatants were filtered using 10 kD filters (Millipore) and spun at 17,000 g for 60 min. To measure NADH, NAD^+ was decomposed by incubation at 65 °C for 30 min. Standard curves (5–200 pg/ml) were generated for quantification.

ATP detection

Twenty milligrams of mice cerebellar samples or 4 X 10^5 cells were harvested, and ATP levels were measured with a commercially available ATP Assay Kit (Colorimetric/Fluorometric) (Abcam, ab83355) according to the manufacturer's protocol.

RNA purification

Twenty milligrams of mice cerebellar cortex or 4 X 10^5 cell pellets were homogenized and RNA was extracted purified with PureLink™ RNA isolation kit following manufacturer's protocol (Thermo). RNA concentration and purity were conducted using a NanoDrop ND-1000 spectrophotometer.

Microarray

The quality of the RNA was inspected using a 2100 Bioanalyzer (Agilent Technologies). Samples with RNA integrity less than 7.5 were discarded. The microarray was performed by the Gene expression and Genomics core facility (NIA) and analyzed using DIANE 6.0 software as described before. A gene was considered significant if the absolute value of its absolute value of z-ratio ≥1.5, p-value ≤ 0.05, false discovery rate ≤ 0.30, as well as sample group independent ANOVA test p ≤ 0.05. A complete set of 880 canonic pathways and 2392 chemical perturbation gene sets were obtained from the Molecular Signatures Database (MSigDB, Broad Institute, Massachusetts Institute of Technology, MA, USA). The complete set was tested for Geneset enrichment using parametric analysis of gene set enrichment (PAGE). Raw microarray hybridization intensity data were filtered to remove undetected and array control probes, before computing log z-score for each sample to identify and exclude possible outlying samples via clustering, scatter plots, and principal components analysis. In A-T fibroblasts, we excluded HT5 and AT5 based on above criteria. After removal of the outlying samples, a sample-specific quantile normalization of the filtered raw average signal was employed to yield quantile normalized log scale z-scores. These quantile z-scores were used to ensure a standard normal distribution in further statistical analysis, including ANOVA, t- and z testing. |Z-ratio| ≥1.5 were used as cut-off values and calculated using a combination of thresholds, including z-test p < value 0.05, FDR ≤ 0.30, and average signal intensity of comparison>0, as well as global sample ANOVA F-test p ≤ 0.05. The gene expression change z-ratio values were then used as input to perform PAGE testing. For each gene set change, an aggregate z-score, and p-value with false discovery change was calculated and reported for statistical significance. The accession number for the raw and processed microarray data reported in this paper is GSE152289.

Quantitative real-time PCR

cDNA was synthesized using iScript cDNA Synthesis kit (BioRad) and qPCR analysis was done with power SYBR Green PCR master mix (Thermo Fisher). The primers used to amplify each transcript are as Table S2.

Immunofluorescence

Anesthetized mice were first perfused with cold PBS and then with 4% paraformaldehyde (PFA) in PBS. The collected brains were then placed in 30% sucrose in PBS (Sigma) until sunken at 4 °C. 1:8 series equidistant floating 30-μm coronal sections (interval 240 μm) were obtained with a cryostat CM 3080S (Leica). Approximately 9-10 slices of each mouse were incubated in blocking buffer (5% goat serum and 0.3% Triton X-100 in PBS) for 60 min at room temperature (RT). Thereafter, brain slices were incubated overnight with the primary antibody at 4°C and then incubated with the appropriate fluorescent probe conjugated secondary antibodies for 1 h at RT protected from light in a humid box. Nuclei were stained with DAPI at 1:5000 dilution for 20 min. The pictures were taken using a Zeiss 880 LSM confocal microscope (Zeiss).

Antibodies

Specific primary antibodies used include rabbit anti-GFAP (DAKO, Z033401-2), goat anti-IBA1 (NOVUS, NB100-1028), mouse anti-dsDNA (Sigma, MAB1293), rabbit anti-TOMM20 (Proteintech, 11802-1-AP), mouse anti-NeuN (Millipore, MAB377), rabbit anti-ATM (Abcam, ab201022), mouse anti-IL1β (Cell signaling, 12242S), rabbit anti-IL6 (Cell signaling, 12912S), rabbit anti-IFNγ (Proteintech, HZ-1301), rabbit anti-P16 (Cell signaling, 92803), rabbit anti-β-actin (Cell signaling, 8457S), Acetyl-p53 (Cell signaling, 2570S), Rabbit anti-Cleaved-Caspase3 (Cell signaling, 9664L), rabbit anti-*p*-STING (Cell signaling, 19781S), rabbit anti-STING (Cell signaling, 13647S), rabbit anti-*p*-P65 (Cell signaling, 3033S), rabbit anti-P65 (Cell signaling, 8242S), mouse anti-NLRP3 (Adipogen, #AG-20B-0014-C100), rabbit anti-pAMPK (Thr172) (Cell Signaling, 2535S), rabbit anti-AMPK (Cell Signaling, 5831), mouse anti-α-tubulin (Thermofisher, A11126), rabbit anti-53BP1 (Cell signaling, 4937S), rabbit anti-CLPP (Proteintech, 15698-1-AP), rabbit anti-HSP60 (Proteintech,15282-1-AP), rabbit anti-PINK1 (Proteintech, 23274-1-

AP), rabbit anti-Parkin (Proteintech, 14060-1-AP), mouse anti-BCL2 (Santa cruz, sc-7382), rabbit anti-*p*-P62 (Proteintech, 39786S), rabbit anti-P62 (Proteintech, 23214S), rabbit anti-VDAC (Proteintech, 10866-1-AP), rabbit anti-NIX (Cell signaling, 12396S), mouse anti-γH2AX (Millipore, 05-636-I), mouse anti-P21 (Novus, NBP2-29463), rabbit anti-LC3 (Novus, NB100-2220).

Respirometry on cells or primary neurons

4 X 10^4 cells or neurons per well were seeded on Seahorse culture plate. Basal and uncoupled oxygen consumption rates (OCRs) and the extracellular acidification rate (ECAR) were measured using the Seahorse extracellular flux bioanalyzer (XF96, Seahorse Bioscience Inc.. To uncouple mitochondria, 2 μM of FCCP was injected after a basal respiration measurement. All measurements were performed in 4 or 5 replications and results were normalized to protein level.

Western blotting

Western blotting was performed according to manufacturer's instructions. Briefly, cell pellets or tissue were lysed using 1x RIPA buffer (Sigma-Aldrich, R0278-50ML) containing protease inhibitor cocktail (Sigma-Aldrich, P8340-5ML) and phosphatase inhibitor cocktail 1 (Sigma-Aldrich, P2850-5ML). Protein concentration were measured with pierce™ BCA protein assay kit (Thermo fisher, 23225). 20 μg samples were loaded and separated on 4-12% Bis-Tris gel (Thermo Fisher Scientific, #NP0336BOX) and transferred to PVDF membranes. Membranes were blocked in TBST + 5% milk, incubated overnight with primary antibody, washed in TBST, incubated with HRP-conjugated secondary antibody for 60 min. Immunoreactivity was detected with an ECL kit (Millipore, or Thermo). Optical density of the immunoreactivity bands was analyzed using Image J software (NIH). All secondary antibodies were obtained from Cell signaling.

SA-β-galactosidase assay

Senescence-associated β-galactosidase (SA-β-gal) activity was detected using the senescence β-galactosidase staining kit (Cell signaling), according to manufacturer's instructions. For cerebellar slices, staining for SA-β-Gal was performed on free-floating

mouse brain sections in the SA-β-gal staining solution at 37°C for 16–20 h. For cells, cells were fixed with 4% formaldehyde in PBS for 15min at room temperature. The fixed cells were incubated with SA-β-Gal staining solution at 37 °C for 16 h after PBS washing. The percentages of positively stained cells were calculated based on three replicates.

Rat neurons culture and treatments

Cerebral cortex tissue was collected from Sprague-Dawley rat embryos at 18 days of gestation. Dissociated cells were seeded into polyethyleneimine-coated plastic dishes or slide chambers in Neurobasal Plus Medium (Thermo fisher, A3582901) supplemented with 2% B27 Plus Supplement (Thermo fisher Scientific, A3582801) and 1% Anti-Anti (Gibco) at a density of 80,000 cells/cm^2. The neurons were grown in 20% O2 + 5% CO2 at 37°C for further experiments. Rat cortical neurons at 0 day in vitro (DIV) were treated with either DMSO or 1µm ATM inhibitor KU-60019 (Sigma, SML1416-25MG) then treated for 72 h, then change with fresh medium with treatment on 3 DIV and 6 DIV. On 6 DIV, neurons were treated with dH_2O or Nicotinamide riboside/NR, 1mM. Then on 7 DIV the assays for the multiple endpoints were performed.

Mitochondrial parameters assay

For A-T cells and human SH-SY5Y were incubated with different dyes (all from Life Technologies ™), including TMRM (40 nM for 15 min) to detect mitochondrial membrane potential, MitoTracker Green (50 nm for 15 min) for mitochondrial content, and mitoSOX (3 µM for 30 min) for mitochondrial ROS, followed by detection by flow cytometer (BD LSRFortessa™). Median number was recorded and analyzed by FlowJo™ v10.6.2.

Subcellular fractionation

Subcellular fractionation and mitochondrial DNA quantification was performed as follows: $5x10^6$ HT1/AT1 or $8x10^6$ SH-SY5Y cells were lysed in 1ml Mitochondrial isolation buffer (225 mM Mannitol, 75 mM Sucrose, 5 mM HEPES, 1 mg/ml Fatty acid free-BSA, Protease and phosphatase inhibitors, adjust pH 7.4). Samples were

centrifuged at 2,200xg for 3 min at 4°C. Pellet are nuclear fractions. Supernatants were transferred to fresh tubes and centrifuged at 17,000xg for 10min at 4C, transferring supernatants to fresh tubes between centrifugation steps to finally yield cytoplasmic fractions. 10 ng cytoplasmic DNA was used for qPCR analysis of mitochondrial DNA using gene-specific primers; nuclear gene RPL13A was quantified from the respective 10ng from nuclear fraction for normalization. Primers are shown as Table S2.

Electron microscopic studies

For electron microscopic studies in mice, sixteen (n = 4 mice per group) mice were deeply anesthetized with chloral hydrate (35 mg per 100 g), and perfused transcardially with 4% (wt/vol) PFA with 2.5% (vol/vol) glutaraldehyde and 15% (vol/vol) picric acid in 0.1 M phosphate buffer (PB, pH 7.3). Brains were left in this fixative solution for 2 h at 4 °C, solution was replaced with 2% PFA and left overnight at 4 °C. Brains were rinsed with PBS, and cut into coronal serial sections (40 μm thick) with a vibratome (VT1000S, Leica Biosystems). Vibratome brain sections were rinsed with 0.1 M PB (pH 7.3), incubated with 1% (wt/vol) sodium borohydride in PB for 30 min to inactivate free aldehyde groups, rinsed in PB, and then post-fixed with 1.5% glutaraldehyde for 10 min on the shaker at room temperature. Next, after rinsed in PB, sections were fixed with 0.5% (vol/vol) osmium tetroxide for 25 min, and contrasted in 1% (wt/vol) uranyl acetate for 30 min. Sections were dehydrated through a series of graded ethanol (30%, 50%, 70%, 90%, 100%, 100%, and 100%) for 10 min each and with propylene oxide for 10 min to remove the residual ethanol, and flat embedded in Durcupan ACM epoxy resin (14040; Electron Microscopy Sciences). Sections were polymerized in the plastic sandwich units in a 60°C Blue M oven for 48 hr. Sections of 65 nm were cut with an ultramicrotome UC7 (Leica Microsystems), and collected on formvar-coated grids and counterstained with uranyl acetate and lead staining. Serial ultrathin sections of the cerebellum from 16 mice were screened under a Tecnai G2 12 transmission electron microscope (TEM; Thermo Fisher Scientific) equipped with a digital OneView camera (Gatan).

Statistical analysis

The sample size (n) for each experimental group is described in each figure legend. Either Statview 5.0 or GraphPad Prism software was used for statistical analyses. Quantitative data displayed as histograms are expressed as means ± standard error of the mean or means ± standard deviation (represented as error bars). Results from each group were averaged and used to calculate descriptive statistics. Student's t test was used for comparisons between two groups. Statistical significance was set at a P value of $^{*}p < 0.05$, $^{**}p < 0.01$ and $^{***}p < 0.001$.

Supplementary figures and tables

Fig. S1 Senescence phenotypes in ATM deficient cells.

Fig. S2 Mitochondrial dysfunction in ATM deficient cells.

Fig. S3 NR downregulates SASP through PINK1 mediated mitophagy.

Fig. S4 NR improves mitochondrial function and DNA repair in neural cells.

Fig. S5 NR normalizes gene expression in *Atm*$^{-/-}$ mice.

Fig. S6 NR downregulates senescence and SASP in *Atm*$^{-/-}$ mice.

Fig. S7 NR improves mitochondrial functions in *Atm*$^{-/-}$ mice.

Fig. S8 NR enhances motor functions in *Atm*$^{-/-}$ mice.

Table S1 Genes with the largest enrichment score in M. musculus.

Table S2 List of primers used in M. musculus and human cells.

Supplemental Figures

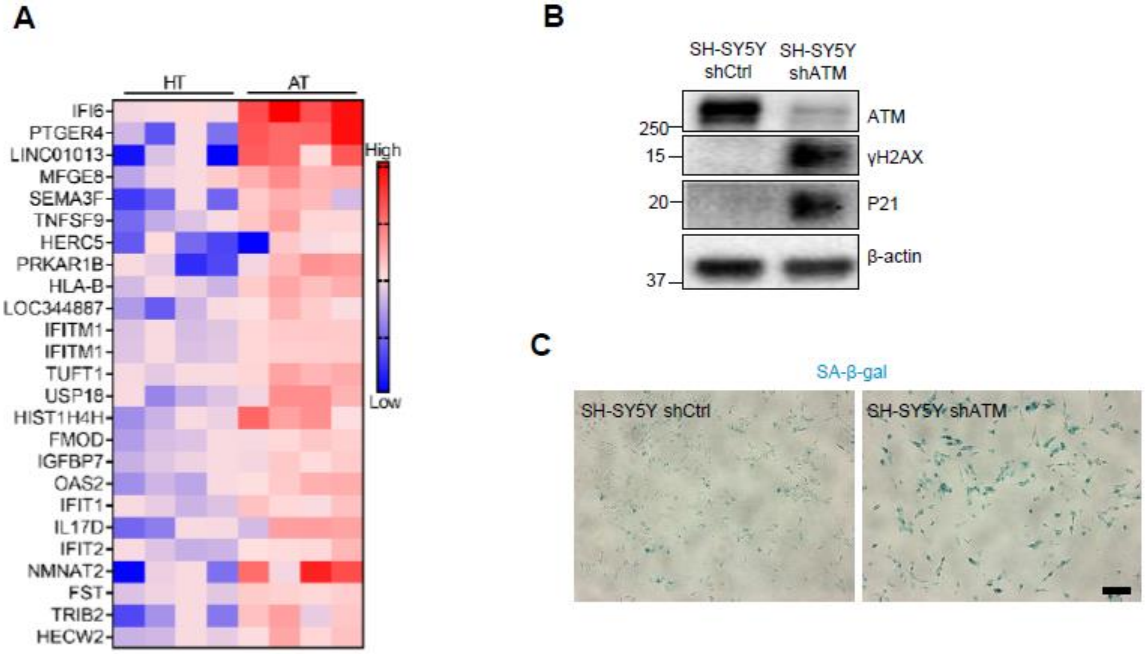

Fig. S1 Senescence phenotypes in ATM deficient cells. (A) Fold-change of top 25 upregulated genes in AT1-4 compare to HT1-4 cells. (B) Immunoblotting analysis of the levels of ATM, *p*-γH2AX, and P21 protein expression in SH-SY5Y shCtrl and shATM cells. (C) Representative images of SA-β-gal staining of SH-SY5Y shCtrl and shATM cells. Scale bar, 100μm.

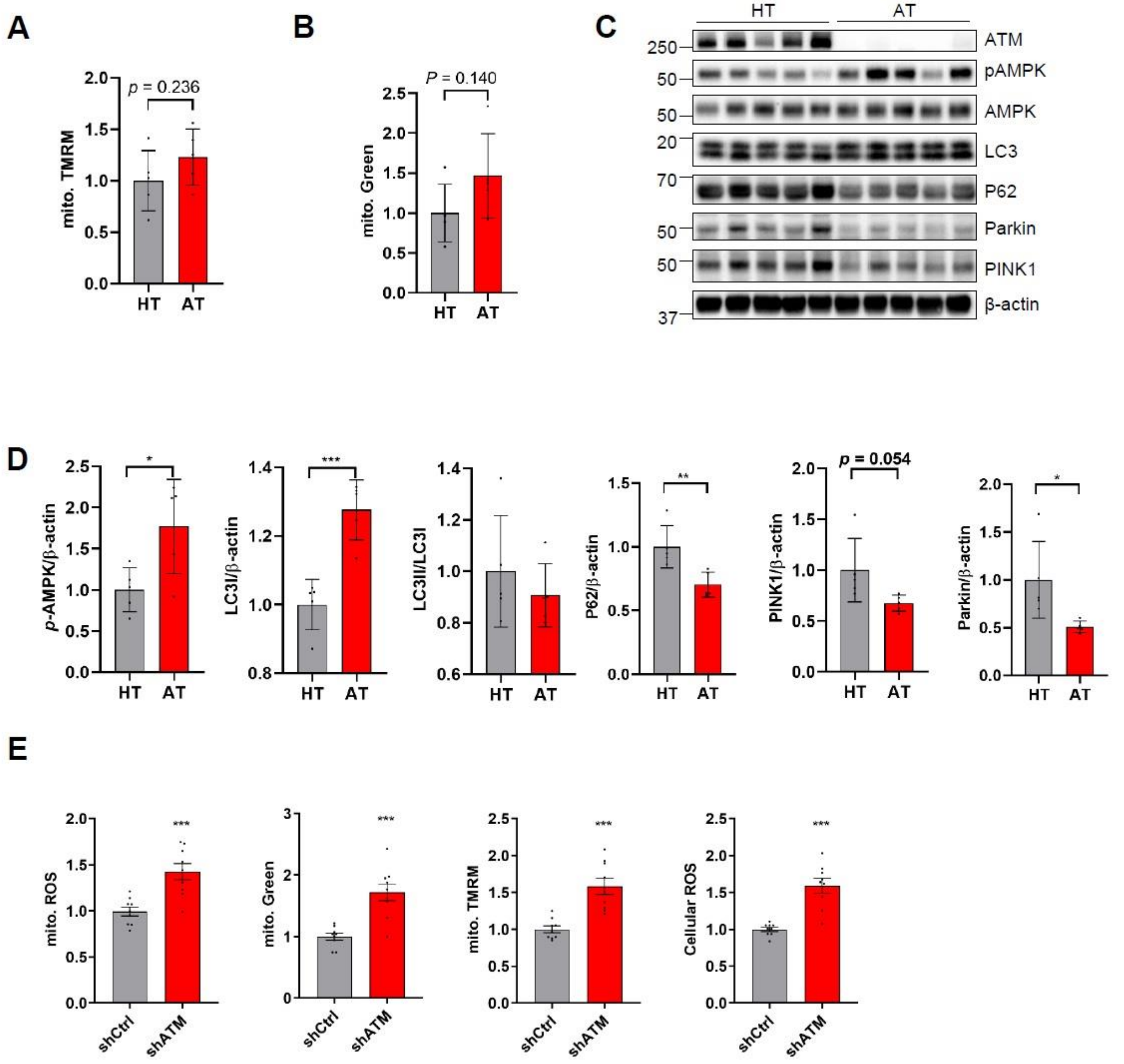

Fig. S2 Mitochondrial dysfunction in ATM deficient cells. (A-B) Mitochondrial membrane potential (mito. TMRM, A) and mitochondrial content (mito. Green, B) were measured by flow cytometry in HT and AT cells. n = 3 cell cultures each group. (C-D) Immunoblotting analysis of the levels of mitophagy markers, including ATM, p-AMPK, LC3, P62, Parkin and PINK1 protein expression in HT and AT cells (C). Quantification of protein expression levels in Fig. S2C (D). (E) Mitochondrial ROS, mitochondrial content, mitochondrial membrane potential, and cellular ROS were measured by flow cytometry in SH-SY5Y shCtrl and shATM cells. n = 8-9 cell cultures in each group. Data are shown as mean ± SD. *$p < 0.05$; **$p < 0.01$; ***$p < 0.001$ by two-tailed Student's t test.

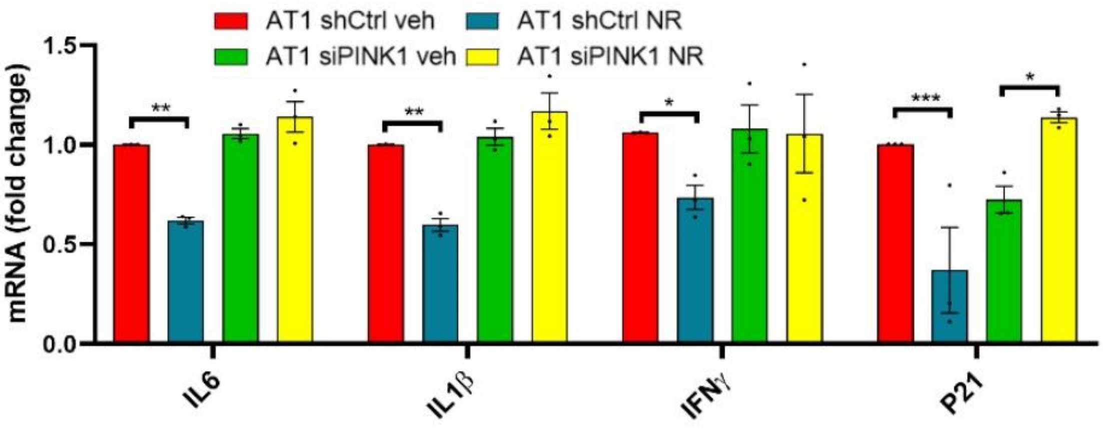

Fig. S3 NR downregulates SASP through PINK1 mediated mitophagy. qPCR analysis of the levels of IL6, IL1β, IFNγ and P21 gene expression in AT1 cells with siCtrl, siPINK1 or NR treatment. n = 3 cultures per group. Data are shown as mean ± SEM. *p < 0.05; **p < 0.01; ***p < 0.001 by one-way ANOVA.

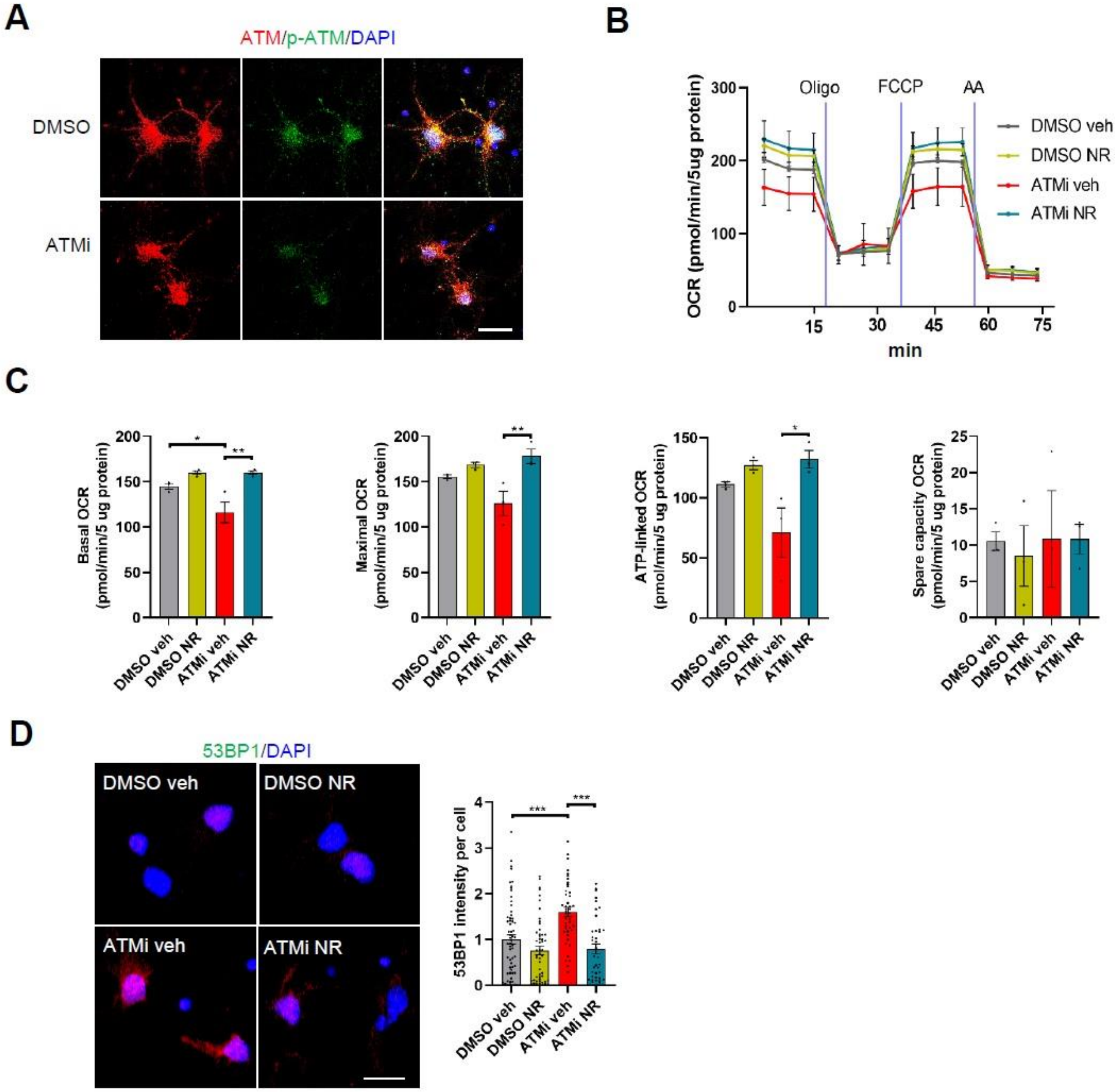

Fig. S4 NR improves mitochondrial function and DNA repair in neural cells. (A) Representative images show the inhibition of ATM kinase activity by ATMi (KU-60019) in primary rat neurons. ATM kinase activity was measured by the phosphorylation ATM (*p*-ATM, green) and ATM protein (red). (B) OCR trace in DMSO or ATMi treated neurons. (C) Quantification of basal, maximal, ATP-linked and spare capacity OCR. n = 3 per group. Data are normalized to protein levels. (D) The representative images of DNA double-strand break marker 53BP1 (left) and quantification of density of 53BP1 (right) in primary rat neurons. Scar bar, 20 µm; 47 – 54 neurons were analyzed for each group. Data are shown as mean ± SEM. *p < 0.05; **p < 0.01; ***p < 0.001 by one-way ANOVA.

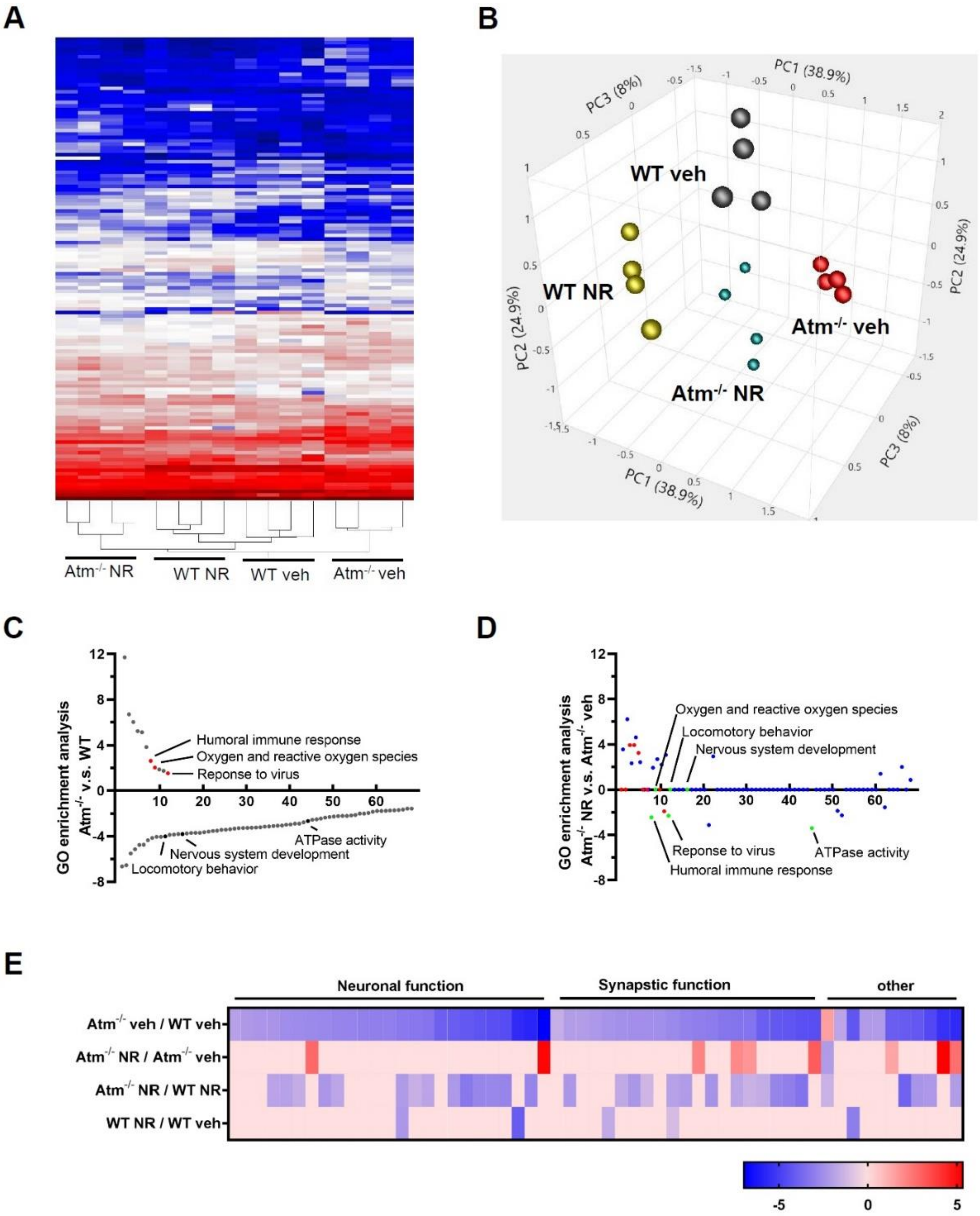

Fig. S5 NR normalizes phenotypes of gene expression in *Atm*$^{-/-}$ mice. (A) Heatmap of differentially expressed genes as determined by microarray of cerebellar tissue. (B) Principle component analysis (PCA) revealed separation between WT veh and *Atm*$^{-/-}$ veh, while NR treatment lead to a normalization of the *Atm*$^{-/-}$ transcriptomic profile closer to the WT veh. (C-D) Go term analysis demonstrates up- and downregulated signaling pathways in *Atm*$^{-/-}$ mice, compared to WT mice. Specific pathways are highlighted, upregulated pathways in red, downregulated pathways in dark (C). Go term analysis shows effect of NR on changed pathways. Specific pathways are highlighted as green, upregulated pathways in Fig. S5C are shown in red, downregulated pathways in Fig. S5C are shown in blue (D). X- axis is pathway number. (E) Neuronal GO term analysis. n = 4 per group.

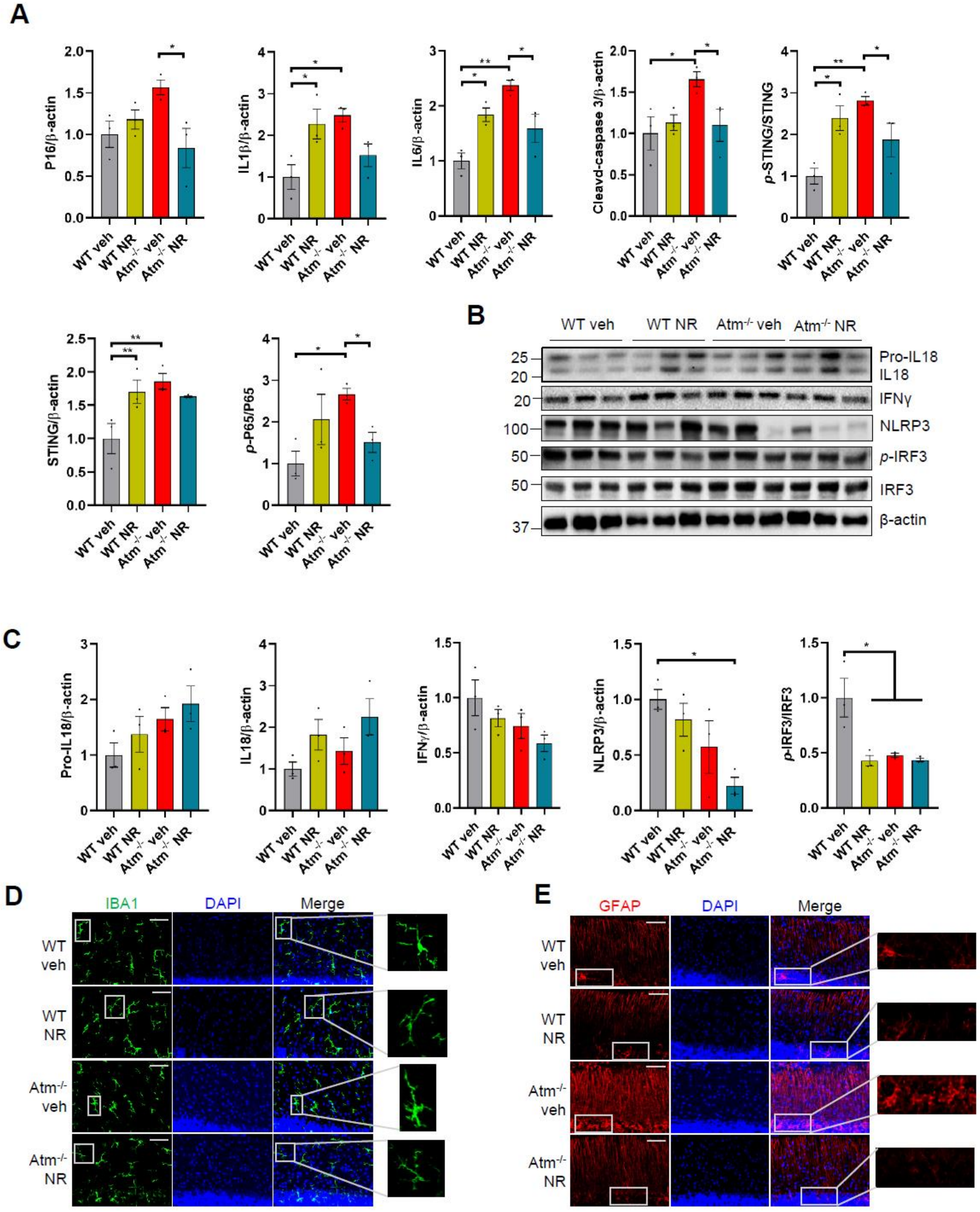

Fig. S6 NR downregulates senescence and SASP in *Atm*$^{-/-}$ mice. (A) Quantification of ATM, P16 (WT veh v.s. *Atm*$^{-/-}$ veh, p =0.058), IL1β (*Atm*$^{-/-}$ veh v.s. *Atm*$^{-/-}$ NR, p = 0.071), IL6, Cleaved-caspase3 (Cl-caspase3), *p*-STING/STING, STING, and *p*-P65/P65 protein levels in Fig. 5F. n = 3 mice per group. (B) Pro-IL18, IL18, IFNγ, NLRP3, and *p*-IRF3/IRF3 were analyzed by immunoblotting in cerebellar tissue. (C) Quantification of protein levels are shown in Fig. S6B. (D) Immunofluorescence with microglia marker

anti-Iba1 (green) antibody in cerebellum. Scale bar, 50 µm. (E) Immunofluorescence with activated astrocytes marker anti-GFAP (red) antibody in cerebellum. Scale bar, 50 µm. Data are shown as mean ± SEM. *$p < 0.05$; **$p < 0.01$; ***$p < 0.001$ by Two-way ANOVA.

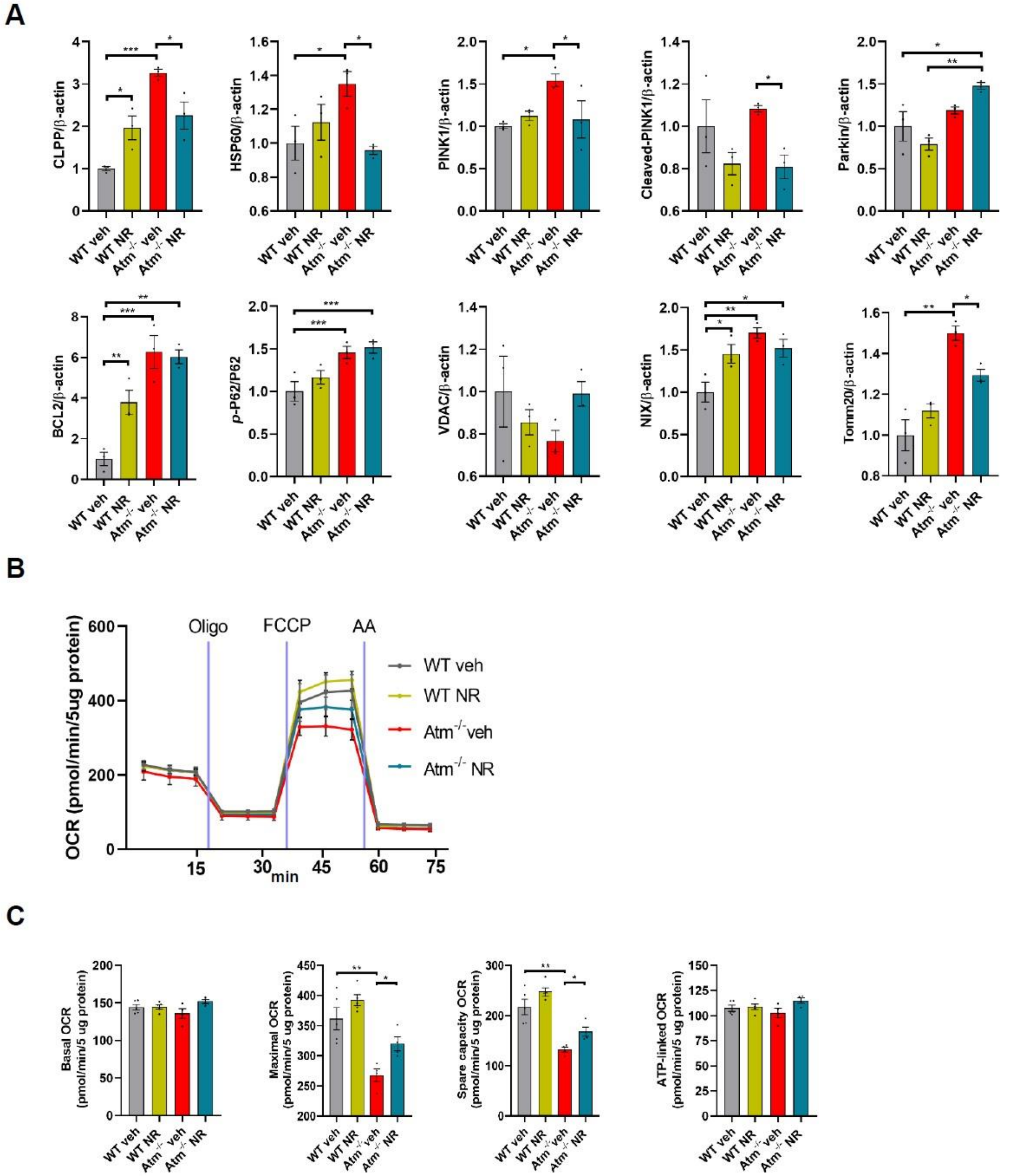

Fig. S7 NR improves mitochondrial functions in *Atm*$^{-/-}$ mice. (A) Quantification of CLPP, HSP60, PINK1, Cleaved-PINK1, Parkin, BCL2, *p*-P62/P62, VDAC, NIX, and Tom20 protein levels in Fig. 7F. n = 3 mice per group. (B) OCR trace in mouse embryo fibroblasts (MEFs) from WT and *Atm*$^{-/-}$ mice. (C) Quantification of basal, maximal, spare respiratory capacity and ATP-linked OCR. Data are normalized to protein levels. N = 4-5 per group. Data are shown as mean ± SEM. *p < 0.05; **p < 0.01; ***p < 0.001 by two-way ANOVA.

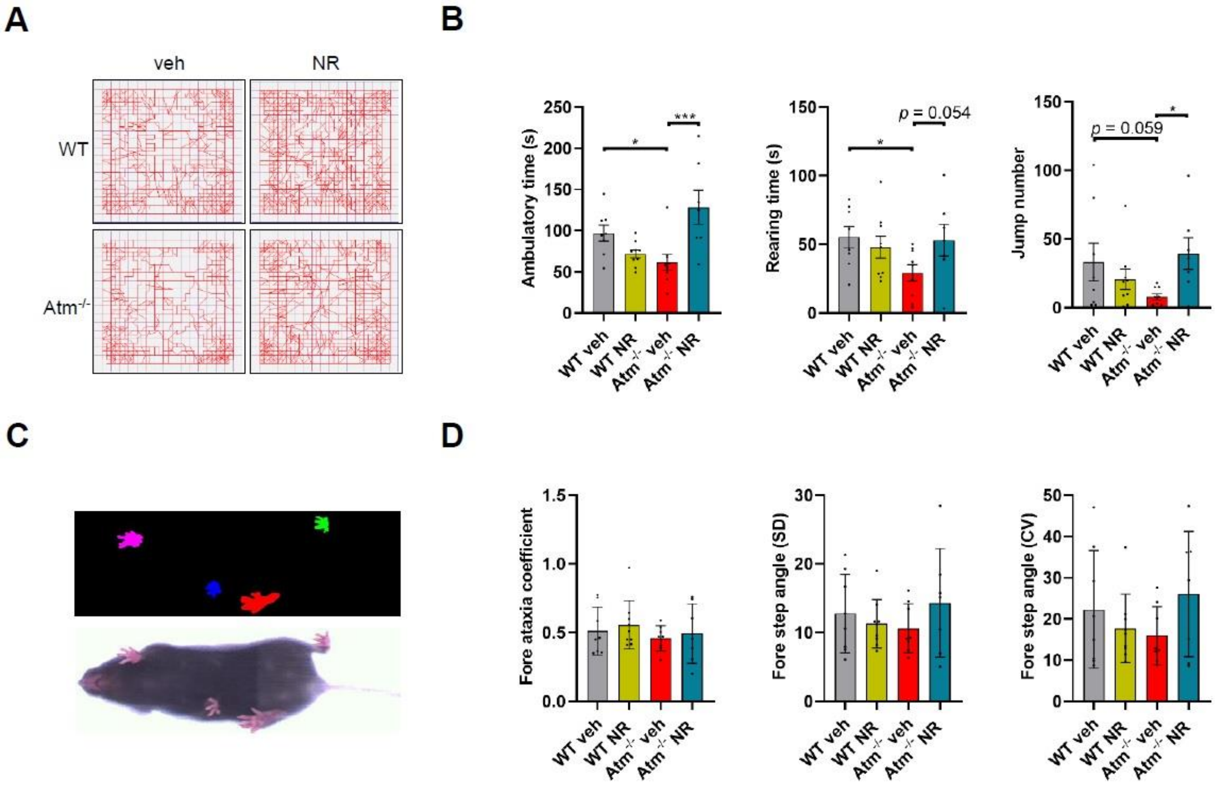

Fig. S8 NR enhances motor functions in *Atm*$^{-/-}$ mice. (A) Representative images of the open-field test in vehicle- or NR-treated mice. (B) Ambulatory time, rearing time, and jump number in the open-field test. (C) Representative images of the Digi-gait test in vehicle- or NR- treated mice. (D) Fore step parameters in the Digi-gait test. WT veh, n = 9; WT NR, n = 10; *Atm*$^{-/-}$ veh, n = 6; *Atm*$^{-/-}$ NR, n = 7. Data are shown as mean ± SEM. *$p < 0.05$; **$p < 0.01$; ***$p < 0.001$ two-way ANOVA.

Supplemental Tables

Table S1 Genes with the largest enrichment score in M. musculus.

Genes	$Atm^{-/-}$ NR / $Atm^{-/-}$ veh		$Atm^{-/-}$ NR / WT NR		$Atm^{-/-}$ veh / WT veh		WT NR / WT veh	
	z ratio	p value	z ratio	p value	z ratio	p value	z ratio	p value
Cpxm2	3.56	0.0026	3.22	0.0226	-1.08	0.1013	0.19	0.7124
Igsf1	2.63	0.0003	-0.3	0.8094	-2.73	0.0007	1.2	0.3057
Klhl1	2.25	0.0015	-0.5	0.6672	-0.78	0.0782	2.67	0.0026
Fam216b	2.18	0	1.72	0.0199	-0.16	0.8472	0.82	0.3581
Chrnb4	2.13	0.0021	0.45	0.5316	-0.12	0.8578	2.1	0
Tmem194	2.09	0.0062	2.03	0.0199	-0.62	0.316	-0.03	0.9505
Car8	2.02	0	0.25	0.0353	0.93	0.2776	3.11	0
Nlrp2	1.9	0.0015	1.15	0.011	0.31	0.7899	1.48	0.0782
Car8	1.86	0	0.29	0.105	1.11	0.3117	3.03	0.0008
A230001M10Rik	1.76	0	0.21	0.821	-1.49	0	0.67	0.5042
4930412C18Rik	1.76	0	0.35	0.1469	1.14	0.2514	2.88	0.0014
Atp6v1g3	1.58	0.0001	0.49	0.2832	0.1	0.8709	1.58	0.0021
Adcy10	1.58	0.0009	1.03	0.0314	-0.13	0.8722	0.82	0.1881
4933409K07Rik	1.57	0.0006	0.06	0.85	-0.91	0.3305	1.11	0.1003
Gpr17	1.56	0	-0.72	0.0136	-2.38	0.0124	0.59	0.4854
E130202H07Rik	1.56	0	0.19	0.6322	0.77	0.3627	2.46	0.0007
Sall1	1.54	0.0017	-0.12	0.8518	-1.63	0.0058	0.62	0.3084
1700011M02Rik	1.53	0	0.6	0.1444	-0.28	0.72	1.05	0.1242
Itga9	1.53	0.0132	-0.07	0.8782	-0.97	0.2292	1.14	0.0031
Fign	1.51	0	-0.37	0.446	-1.56	0	0.9	0.0744
Cfap69	1.47	0	0.23	0.381	-1.62	0.0306	0.18	0.7684
Fam170b	1.46	0.0064	0.87	0.0182	0.49	0.6425	1.37	0.069
Cdkl2	1.37	0.0001	-0.03	0.9434	-1.59	0.0016	0.36	0.4883
Rnf138rt1	1.37	0.0011	0.11	0.8666	-1.79	0.0001	0.02	0.9734
8030453O22Rik	1.36	0.0154	0.32	0.6089	0.11	0.8761	1.48	0.0069
Col1a1	1.34	0.0159	1.54	0.0317	-1.04	0.0008	-0.83	0.0242
Defb45	1.33	0.0018	1.49	0.037	1.45	0.0516	1.41	0.0915
Ccdc173	1.33	0	0.05	0.792	-1.52	0.0005	0.28	0.2527
Hhip	1.33	0	-0.39	0.1584	-1.59	0	0.67	0.0058
Rbms3	1.32	0	-0.34	0.4412	-1.52	0	0.66	0.1142
Fscn3	1.31	0.0035	1.43	0.0136	0.87	0.1423	0.94	0.1002
Adgrg3	1.29	0.0011	0.4	0.2322	0.77	0.4512	1.9	0.0231
Pgc	1.27	0.0001	0.48	0.1252	0.22	0.7824	1.29	0.0504
Dzip1	1.22	0.0111	1.72	0.0004	1.74	0.039	1.29	0.0539
Tnni3	1.21	0.0636	3	0	1.46	0.1626	-0.3	0.6879
4921531P14Rik	1.21	0.0963	1.47	0.0764	1.42	0.0659	1.27	0.0357
Wdr17	1.14	0	-0.13	0.7487	-1.79	0	-0.02	0.965

Auh	1.13	0.0002	0.43	0.2263	0.72	0.2927	1.61	0.0069
Cenpk	1.12	0.0189	1.53	0.0085	-0.7	0.0028	-0.8	0.0002
Lum	1.11	0.0013	0.42	0.3819	-1.65	0.0024	-0.49	0.359
Speer4c	1.11	0.0581	1.56	0.0299	1.43	0.0929	1.05	0.1696
2310030G06Rik	1.1	0.0004	-1.79	0	-3.08	0	0.5	0.3831
Pcdh17	1.07	0	-0.55	0.2023	-1.73	0.0331	0.38	0.6385
LOC102632383	1.01	0.0203	1.21	0.0098	1.01	0.0791	0.92	0.0373
Syt6	1.01	0.0022	-0.24	0.6477	0.72	0.4759	2.16	0.0258
Slc7a3	0.99	0.1436	-1.7	0.3547	-6.61	0.0004	-2.86	0.2286
9330179D12Rik	0.98	0.0293	-0.57	0.6237	-3.87	0	-1.61	0.1955
4932412D23Rik	0.97	0.0853	1.36	0.0197	1.6	0.0278	1.23	0.0183
Tpsb2	0.97	0.1505	1.47	0.0262	1.61	0.1061	1.13	0.1181
Casp14	0.95	0.0267	1.53	0.0023	0.68	0.3064	0.22	0.6967
St8sia4	0.94	0.0001	-0.52	0.1792	-1.52	0	0.37	0.376
Slc44a5	0.89	0.0024	0.15	0.6778	-2.32	0	-1.09	0.0141
Pts	0.89	0.0002	-2.21	0	-3.23	0	0.55	0.1476
4930512M02Rik	0.87	0.0156	1.35	0.0096	1.5	0.0114	1.02	0.0885
Gprc5b	0.81	0.0184	-0.4	0.3783	-1.63	0.0069	-0.02	0.975
Tnnc2	0.8	0.0232	1.9	0	1.08	0.2275	0	0.9995
LOC105243194	0.8	0.0771	1.2	0.0182	1.24	0.0663	0.87	0.1138
Bend4	0.75	0.0077	-0.19	0.5232	-1.72	0	-0.39	0.1411
Gm4470	0.74	0.23	1.25	0.035	1.78	0.0571	1.22	0.0753
Adam32	0.74	0.0005	-0.22	0.6479	0.4	0.3333	1.53	0.0072
Dnaja4	0.73	0	-0.88	0	-1.84	0	0.2	0.2707
E2f1	0.7	0.0417	-0.37	0.2939	-1.59	0.0133	-0.15	0.7798
Slc5a11	0.67	0.0044	-0.36	0.3451	0.44	0.4824	1.61	0.0086
Slc34a3	0.67	0.0239	-1.73	0.0067	-1.43	0.0825	1.37	0.1326
Mex3a	0.65	0.0241	-0.31	0.109	-1.52	0.007	-0.21	0.6044
Olfr1402	0.64	0.1896	1.06	0.03	1.1	0.0719	0.69	0.0986
LOC102637947	0.62	0	1.51	0	0.69	0.0537	-0.16	0.6979
Gm32819	0.6	0.5081	4.14	0	3.02	0.0268	-0.85	0.2379
Oaf	0.58	0.0063	-0.38	0.2883	-1.81	0.0011	-0.48	0.3921
Mast4	0.25	0.668	-2.24	0.0014	0.18	0.787	2.8	0
Pstpip2	0.2	0.461	-1.57	0.0288	-0.04	0.9241	1.84	0.0172
Fxyd6	0.11	0.7871	-2.48	0	-3.57	0	-0.47	0.2035
Rpp25	0.08	0.8789	-2.49	0.0051	-2.13	0.0009	0.8	0.3352
Cryab	0.05	0.8527	-2.41	0	-2.4	0	0.44	0.3555
Gm5617	-0.17	0.0505	1.42	0	1.71	0.0001	-0.17	0.6775
St14	-0.26	0.1536	0.53	0.2749	1.63	0	0.57	0.2938
Tmem25	-0.28	0.2776	-4.61	0	-4.38	0	0.54	0.1961
D830030K20Rik	-0.32	0.0881	0.98	0.011	1.53	0	-0.05	0.8814
Spaca6	-0.34	0.5268	1.01	0.0557	1.64	0.0032	-0.02	0.9419
Mpzl2	-0.44	0.59	-2.25	0.0042	-2.46	0.007	-0.41	0.4031

1700017B05Rik	-0.44	0.0001	1.46	0	1.88	0.0011	-0.4	0.4303
Neurod6	-0.49	0.0628	0.04	0.7381	-1.23	0.0021	-1.76	0
Lrrc49	-0.54	0.071	2.02	0	2.5	0.0002	-0.55	0.2828
6330416G13Rik	-0.65	0.1233	0.57	0.4212	2.03	0	0.39	0.5258
Mapk13	-0.68	0.067	1.46	0.0004	2.21	0.045	-0.42	0.6609
Nxpe4	-0.71	0.0005	-2.81	0	-2.57	0	-0.26	0.588
Ppp1cc	-0.71	0	0.32	0.3188	1.6	0	0.19	0.6258
Prkcd	-0.71	0.0003	0.26	0.5362	2.27	0	0.85	0.1048
Slc12a7	-0.73	0.1018	-0.29	0.5172	-1.12	0.0615	-1.62	0.0001
Nanog	-0.74	0	0.29	0.4914	1.53	0	0.13	0.789
Gm38485	-0.75	0.0201	0.4	0.0716	1.53	0.0268	0.01	0.9892
Frzb	-0.8	0	0.05	0.8125	-0.91	0.2213	-1.88	0.0045
Bmp6	-0.87	0.0606	0.08	0.885	-0.42	0.4382	-1.54	0.0003
Anpep	-0.91	0.012	-0.12	0.7676	-0.61	0.1968	-1.56	0.0001
Trim3	-0.91	0	0.04	0.8522	1.54	0.0038	0.17	0.7295
R3hdm4	-0.92	0	0.3	0.134	1.54	0.0041	-0.1	0.8101
Cntfr	-0.92	0.0079	-0.37	0.3913	1.6	0.0023	0.65	0.1733
Hmmr	-1.07	0.0847	0.5	0.4476	2.29	0.0028	0.17	0.7675
Cd209d	-1.07	0.0005	0	0.9943	1.62	0.0028	0.09	0.8857
Col6a1	-1.08	0.0094	0.1	0.8383	-0.93	0.3778	-2.29	0.0128
Plagl1	-1.12	0	-0.65	0	-0.6	0.2199	-1.27	0.0042
6430411K18Rik	-1.14	0.0013	-1.12	0.0703	-0.68	0.08	-0.88	0.1279
Gnmt	-1.14	0.0116	0.78	0.1378	1.57	0.0194	-0.85	0.1388
Sap25	-1.22	0	-0.47	0.1781	-0.39	0.177	-1.4	0
Sp140	-1.31	0	-1.49	0	-0.41	0.2083	-0.47	0.0383
Cyp2t4	-1.32	0.0102	0.7	0.3743	2	0.0007	-0.61	0.3908
Cep164	-1.32	0	-1.44	0.0001	-0.55	0.1059	-0.66	0.0551
Nr4a2	-1.34	0	-0.68	0.091	-0.52	0.1746	-1.45	0.0001
Adamts18	-1.43	0.0065	-1.07	0.0148	-0.32	0.6859	-0.97	0.054
Pign	-1.5	0.0096	-0.46	0.4196	2.11	0.0064	0.45	0.3945
Pfn1	-1.51	0	-0.05	0.8672	1.33	0.0014	-0.68	0.0883
Chl1	-1.52	0	-0.14	0.8358	1.38	0	-0.54	0.4151
5830472F04Rik	-1.54	0.0233	0.54	0.5267	2.58	0.0017	-0.21	0.7799
Zfp395	-1.55	0	-0.03	0.8971	1.13	0.0066	-0.92	0.0198
Atp11b	-1.55	0	-0.26	0.4733	0.79	0.0005	-0.99	0
Mybpc1	-1.57	0.0005	0.34	0.6309	1.5	0.026	-1.01	0.1838
LOC102640292	-1.59	0.0001	-0.38	0.3341	1.31	0.0218	-0.44	0.2576
Tfpi	-1.6	0.0186	0.21	0.5164	1.96	0.0477	-0.49	0.1995
Cpne1	-1.62	0.001	-0.15	0.8361	2.26	0.0014	0.13	0.8689
Scn11a	-1.62	0.0108	-0.94	0.1666	0.75	0.0912	-0.4	0.0004
D230040J21Rik	-1.65	0.0111	-0.8	0.234	0.34	0.5659	-0.95	0.0017
Tgfb1i1	-1.65	0.0015	0.1	0.5741	0	0.9974	-2.19	0.0004
Ap2b1	-1.66	0	-0.75	0.1049	1.32	0.0848	-0.15	0.8389

A030005L19Rik	-1.68	0.0007	-0.07	0.9067	1.8	0.0003	-0.45	0.3125
A330023F24Rik	-1.7	0.0003	-0.58	0.2356	0.33	0.605	-1.25	0.0105
Gfap	-1.79	0.0004	-0.29	0.4597	1.45	0.0952	-0.67	0.2614
Syt10	-1.83	0.0313	3.68	0.0009	2.8	0.0139	-3.63	0.001
5730480H06Rik	-1.9	0.0106	-0.67	0.341	1.09	0.1749	-0.74	0.0621
Spock1	-1.93	0	-0.44	0.3085	1.22	0.0001	-0.89	0.0321
Cdkn1a	-1.94	0.0072	-0.13	0.4566	1.72	0.0973	-0.78	0.0006
Cecr5	-2.01	0	-0.19	0.6041	1.03	0.0027	-1.43	0.0005
Fam219aos	-2.25	0.0036	-0.13	0.8351	2.73	0.036	-0.27	0.7624
Gm7420	-2.29	0	-0.47	0.4807	1.83	0.0081	-0.78	0.2321

Table S2 List of primers used in M. musculus and human cells

	Gene Symbol	Gene ID	Forward	Reverse
M. musculus	Gapdh	14433	TGTGTCCGTCGTGGATCTGA	CCTGCTTCACCACCTTCTTGAT
	Cox5a	12858	GAGCCCAAAATCATTGATGC	TGAGGTCCTGCTTTGTCCTT
	Cox2	17709	AACCGAGTCGTTCTGCCAAT	CTAGGGAGGGGACTGCTCAT
	Nd1	17716	CAAACACTTATTACAACCCAAGAACA	TCATATTATGGCTATGGGTCAGG
	Sdhc	66052	GCTGCGTTCTTGCTGAGACA	ATCTCCTCCTTAGCTGTGGTT
	Arg1	11846	GGACCTGGCCTTTGTTGATG	AGACCGTGGGTTCTTCACAATT
	Fizz1	57262	CCCTCCACTGTAACGAAGACTC	CACACCCAGTAGCAGTCATCC
	Ym1	12655	CATGAGCAAGACTTGCGTGAC	GGTCCAAACTTCCATCCTCCA
human cells	GAPDH	2597	TGCACCACCAACTGCTTAGC	GGCATGGACTGTGGTCATGAG
	IL6	3569	GCCCAGCTATGAACTCCTTCT	GAAGGCAGCAGGCAACAC
	IL1β	3553	CTGTCCTGCGTGTTGAAAGA	TTGGGTAATTTTTGGGATCTACA
	TNFα	7124	CAGCCTCTTCTCCTTCCTGAT	GCCAGAGGGCTGATTAGAGA
	IFN-γ	3458	TGACCAGAGCATCCAAAAGA	CTCTTCGACCTCGAAACAGC
	P21	1026	CGAAGTCAGTTCCTTGTGGAG	AGTCGTGGTCTTTGGGAGTC
	P16	1029	CCCAACGCACCGAATAGTTA	ACCAGCGTGTCCAGGAAG
	CCND1	595	ATGTTCGTGGCCTCTAAGATGA	CAGGTTCCACTTGAGCTTGTTC
	CCNA1	8900	GAAATTGTGCCTTGCCTGAGTG	TCTGATATGGAGGTGAAGTTCTGGA
	HSPA9	3313	TGGTGAGCGACTTGTTGGAAT	ATTGGAGGCACGGACAATTTT
	HSP60	3329	GGGTAACCGAAGCATTTCTGC	CTGCACTCTGTCCCTCACTC
	YME1L1	10730	AGGGACCTTGGATTATCTGAACT	TGGGATGTATGCCAATGGGAA
	CLPP	8192	AAGCACACCAAACAGAGCCT	AAGATGCCAAACTCCTGGG
	BNIP3	664	AGGGCTCCTGGGTAGAACTG	CCCTGTTGGTATCTTGTGGTG
	P62	8878	CCTTGCCCTACAGCTGAGTC	CTTGTCTTCTGTGCCTGTGC
	PARK2	5071	TCAATCTACAACAGCTTTTATG	TGCACTAGTCCCAGGGCA
	BECN1	8678	GGCTGAGAGACTGGATCAGG	CTGCGTCTGGGCATAACG
	mt-ND1	4535	CACCCAAGAACAGGGTTTGT	TGGCCATGGGTATGTTGTTAA
	D-LOOP	NC_012920	CTATCACCCTATTAACCACTCA	TTCGCCTGTAATATTGAACGTA
	mt-CO2	4513	AATCGAGTAGTACTCCCGATTG	TTCTAGGACGATGGGCATGAAA
	mt-ATP6	4508	AATCCAAGCCTACGTTTTCACA	AGTATGAGGAGCGTTATGGAGT
	RPL13A	23521	GCCCTACGACAAGAAAAAGCG	TACTTCCAGCCAACCTCGTGA
